# Therapeutic efficacy of rscAAVrh74.miniCMV.*LIPA* gene therapy in a mouse model of lysosomal acid lipase deficiency

**DOI:** 10.1016/j.omtm.2022.08.001

**Published:** 2022-08-04

**Authors:** Patricia Lam, Anna Ashbrook, Deborah A. Zygmunt, Cong Yan, Hong Du, Paul T. Martin

**Affiliations:** 1 Center for Gene Therapy, Abigail Wexner Research Institute, Nationwide Children’s Hospital, 700 Children’s Dr., Columbus, OH 43205, USA; 2 Department of Pathology and Laboratory Medicine, Indiana University School of Medicine, Indianapolis, IN 46202, USA; 3 Department of Pediatrics, The Ohio State University College of Medicine, Columbus, OH 43205, USA

**Keywords:** liver disease, lysosomal storage disorder, lysosomal acid lipase, cholesterol, triglyceride, gene therapy

## Abstract

Lysosomal acid lipase deficiency (LAL-D) presents as one of two rare autosomal recessive diseases: Wolman disease (WD), a severe disorder presenting in infancy characterized by absent or very low LAL activity, and cholesteryl ester storage disease (CESD), a less severe, later onset disease form. Recent clinical studies have shown efficacy of enzyme replacement therapy for both forms of LAL-D; however, no gene therapy approach has yet been developed for clinical use. Here, we show that rscAAVrh74.miniCMV.*LIPA* gene therapy can significantly improve disease symptoms in the *Lipa*^*−/−*^ mouse model of LAL-D. Treatment dramatically lowered hepatosplenomegaly, liver and spleen triglyceride and cholesterol levels, and serum expression of markers of liver damage. Measures of liver inflammation and fibrosis were also reduced. Treatment of young adult mice was more effective than treatment of neonates, and enzyme activity was elevated in serum, consistent with possible bystander effects. These results demonstrate that adeno associated virus (AAV)-mediated *LIPA* gene-replacement therapy may be a viable option to treat patients with LAL-D, particularly patients with CESD.

## Graphical abstract

**Figure:**
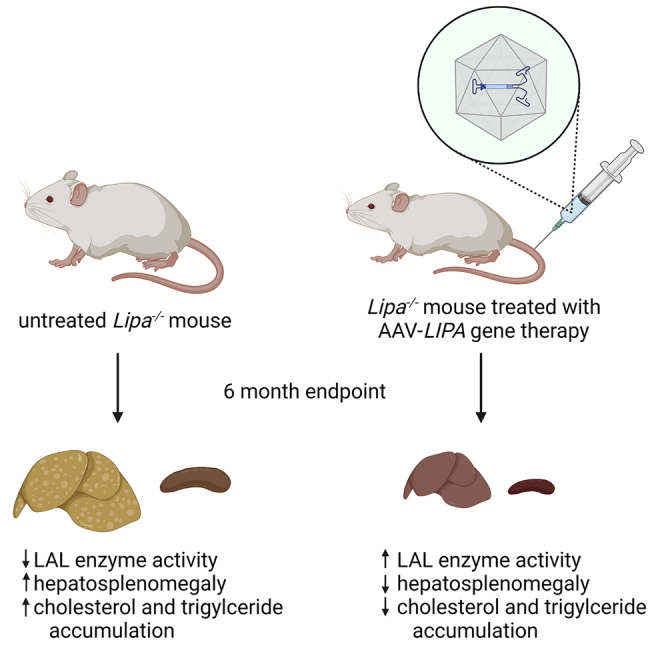

## Teaser

This study developed an AAV-based gene therapy for lysosomal acid lipase deficiency (LAL-D), a rare disorder that results from mutations in the lipase A (*LIPA*) gene. The authors found that liver damage, lipid accumulation, and reduced enzyme activity were corrected by gene replacement in a mouse model of LAL-D.

## Introduction

Lysosomal acid lipase deficiency (LAL-D) is a lysosomal storage disorder caused by mutations in the lipase A (*LIPA*) gene that result in a failure of the LAL enzyme to sufficiently hydrolyze cholesteryl esters and triglycerides into free cholesterol and free fatty acids in the lysosome.^1^, ^2^, ^3^, ^4^ LAL occupies a critical and essential position in the control of plasma lipoprotein levels and in the prevention of cellular lipid overload, especially in the liver and spleen.^2^^,^^3^ LAL-D is a rare genetic disease, with prevalence ranging from 1 in 40,000 to 1 in 300,000, though disease incidence may be underestimated through misdiagnosis as non-alcoholic fatty liver disease (NAFLD) in some instances.^3^^,^^4^ Null *LIPA* gene mutations cause Wolman disease (WD), a fatal disease of infancy named after Moshe Wolman, who reported one of the first cases.^5^ WD is typically characterized by hepatomegaly with liver dysfunction, dyslipidemia (elevated serum triglycerides and low-density lipoprotein (LDL) cholesterol with reduced HDL cholesterol), pulmonary fibrosis, adrenal calcification, and adrenal insufficiency.^2^, ^3^, ^4^^,^^6^^,^^7^ Infants manifest disease in the first month of life and fail to thrive, most likely due to liver disease combined with a failure to absorb nutrients through the intestinal lining. Median lifespan of untreated infants with WD is 3.7 months.^6^^,^^7^ Partial loss of function *LIPA* mutations, usually with 1%–12% of normal activity, give rise to cholesteryl ester storage disease (CESD), a later onset, less severe disease form.^3^^,^^4^^,^^6^^,^^7^ While CESD need not result in premature death, it can be associated with significant morbidity, including hepatosplenomegaly, liver fibrosis, liver cirrhosis, and liver failure.^3^^,^^4^^,^^6^ Chronic dyslipidemia in LAL-D may also cause accelerated atherosclerosis with an associated high risk of cardiac disease, including myocardial infarction, and cerebrovascular complications, including stroke.^3^^,^^4^^,^^6^^,^^7^ Liver biopsy in patients with LAL-D typically demonstrate micro- and macro-vascular steatosis involving Kupffer cells and hepatocytes, accompanied by fibrosis and cirrhosis as the disease progresses.^6^, ^7^, ^8^ Unlike other lysosomal storage disorders such as Gaucher disease and Niemann-Pick disease, there appears to be little CNS involvement.^3^^,^^6^^,^^7^

Lipase A-deficient (*Lipa*^−/−^) mice have been used to demonstrate proof of concept for potential therapies, including enzyme replacement therapy.^9^, ^10^, ^11^, ^12^, ^13^ *Lipa*^−/−^ mice typically live until 6–7 months of age and show LAL-D phenotypes, in particular hepatosplenomegaly, elevated serum aspartate aminotransferase (AST) and alanine aminotransferase (ALT), and elevated liver and spleen cholesterol and triglycerides.^13^ The fact that the mice live about a quarter of their normal lifespan suggests that they more closely mimic CESD than WD. *Lipa*^−/−^ rats were used to help support an investigational new drug application for sebelipase alfa (also known as Kanuma), an enzyme replacement therapy, which has now been shown to have clinical efficacy in patients with WD and CESD and is approved for use by the FDA.^14^, ^15^, ^16^, ^17^, ^18^ Such treatments require protein infusions every 2 weeks and give rise to only partial clinical correction.^19^ In phase 3 enzyme replacement clinical trial of sebelipase alfa, elevations in serum AST/ALT, total bilirubin, and LDL cholesterol were reduced, and there was a 32% overall reduction in liver fat content and a 6.8% reduction in spleen volume.^20^^,^^21^

While enzyme replacement therapy has shown clinical benefit, little work has been done to develop a single treatment gene therapy for LAL-D. Several gene-replacement studies were previously reported using an adenoviral vector that showed clinical benefits,^22^^,^^23^ but no treatment using adeno-associated virus (AAV), the current gold standard for human gene therapy, has yet to be developed. Accordingly, here we have developed a recombinant (r), self-complementary (sc) AAV vector (serotype rh74) expressing the human *LIPA* cDNA under control of the minimal cytomegalovirus (CMV) promoter rscAAVrh74.miniCMV.*LIPA* and tested its therapeutic impact in *Lipa*^−/−^ mice.

## Results

### rscAAVrh74.miniCMV.*LIPA* treatment impacts hepatosplenomegaly and elevated serum transaminases in *Lipa*^−/−^ mice

**Figure 1:**
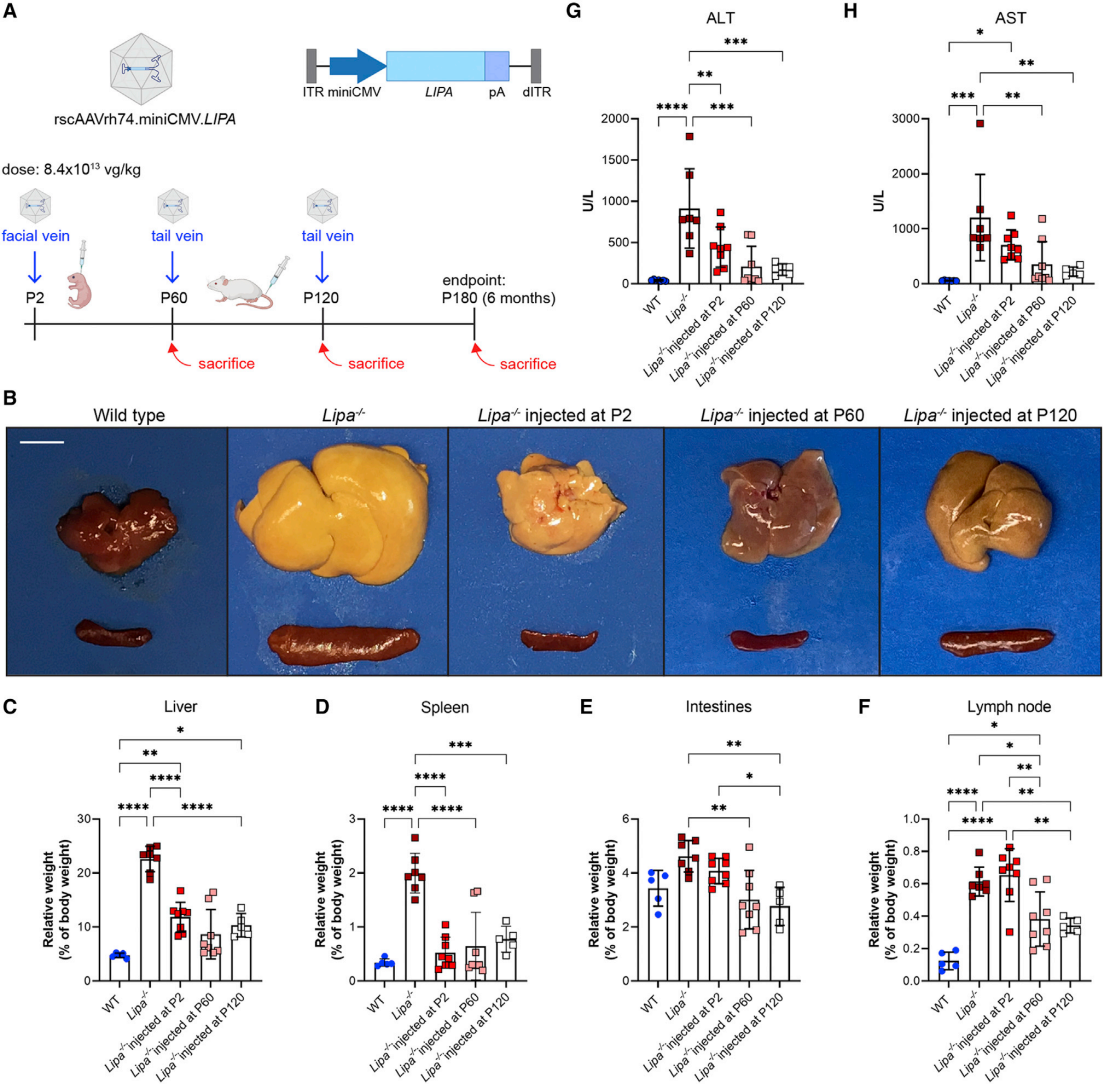
rscAAVrh74.miniCMV.*LIPA* treatment reverses hepatosplenomegaly and elevated serum liver enzymes in *Lipa*^−/−^ mice (A) Schematic of treatment plan of *Lipa*^−/−^ mice. Mice were injected at P2, P60, and P120. Disease progression was followed at time points of 2, 4, and 6 months of age. (B) Gross pathology of liver and spleen from wild-type, untreated *Lipa*^−/−^, and treated *Lipa*^−/−^ mice at 6 months of age. Scale bar: 1 cm. (C–F) Relative weights of liver, spleen, intestines, and mesenteric lymph node at 6 months of age. (G and H) Serum alanine aminotransferase (ALT) and aspartate aminotransferase (AST) levels at 6 months of age. All data represented as mean ± SD (n = 5–8). Statistical significance was defined as p ≤ 0.05 (∗p ≤ 0.05, ∗∗p ≤ 0.01, ∗∗∗p ≤ 0.001, ∗∗∗∗p ≤ 0.0001) using one-way ANOVA with Tukey’s post-hoc test. Schematic created with BioRender.com. ITR, inverted terminal repeats; pA, SV40 polyA; dTIR, mutated ITR.

rscAAVrh74.miniCMV.*LIPA* AAV gene therapy at a dose of 8.4 x 10^13^ vector genomes (vg) per kilogram (vg/kg) was intravenously injected at early (post-natal day 2 [P2]), middle (P60), or advanced (P120) disease stages in *Lipa*^−/−^ mice (Figure 1A). For each injection time point, mice were followed to endpoints of 2, 4, and 6 months of age to assess disease progression. Wild-type (WT) mice were also injected at P2 and P60 with the same AAV dose to observe potential toxicity and other side effects of treatment. Hepatosplenomegaly and discoloration of organs due to increased fat deposition are both defining features of LAL-D and of disease in *Lipa*^−/−^ mice. Both phenotypes were present and progressed with age in *Lipa*^−/−^ mice (Figures 1B–1D and S1A–S1C). Liver weight increased over time to comprise as much as 25% of total body weight by 6 months of age, in contrast to being only 5% of body weight at all 3 ages in WT mice (Figures 1C and S1B). Similarly, spleen weight increased, on average, to 2% of total body weight at 6 months of age in *Lipa*^−/−^ mice compared with 0.3% in WT (Figures 1D and S1C). Treatment of *Lipa*^−/−^ mice at P2, P60, and P120 resulted in reduced liver weight and improved appearance, but early treatment (P2) was far less effective than treatment at P60 or P120. P2 injection reduced liver size to WT levels at 2 and 4 months, suggesting disease inhibition, but only partially reduced liver weight, by about 50%, at 6 months. By contrast, P60 and P120 injections were more effective despite already present evidence of hepatosplenomegaly at these ages (Figures S1A and S1B), reducing liver weight to 8% and 10% of total body weight, respectively, at 6 months. P2 injection was more effective at reducing spleen size than it was for liver at the 6-month endpoint (Figure 1D), and treatment at all 3 time points reduced spleen weight to near WT levels (Figure S1C). Intestines and mesenteric lymph nodes also showed increased weight in *Lipa*^−/−^ mice (Figures 1E, 1F, S1D, and S1E). Weight in intestine was increased to 4.5% of total body weight at 6 months of age compared with 3% in WT (Figure 1E), while weight of mesenteric lymph node was increased to 0.6% of body weight compared with 0.1% in WT (Figure 1F). Here again, AAV treatment reduced intestine and mesenteric lymph node weight in a manner similar to responses seen with liver, with P2 injection showing improvement to near WT levels at 2 or 4 months that was lost by 6 months, while injection at P60 or P120 showed reductions in weight at the 6-month endpoint (Figures S1D and S1E). Unlike liver, spleen, and intestine, there were no data that showed near normalization of lymph node weight at any time point with any of the treatment times tested. Instead, at best, only a 50% average reduction in weight was achieved by 6 months (Figures 1F and S1E).

We also measured serum ALT and AST activity at 2, 4, and 6 months of age. These enzyme measures may, when elevated, indicate liver damage (Figures 1G, 1H, S2A, and S2B). Both serum ALT and AST levels were elevated 20-fold in *Lipa*^−/−^ mice compared with WT at the 6-month endpoint. Here, treatment at all time points resulted in decreased serum ALT/AST levels, with a more pronounced effect with injection at P60 and P120 than at P2 (Figures S2A and S2B). As with liver weights, injection at P2 significantly reduced serum ALT and AST levels at the 2- and 4-month time points to near WT levels but showed only about a 50% reduction at 6 months (Figures S2A and S2B). Injection of this dose of rscAAVrh74.miniCMV.*LIPA* in WT mice did not significantly elevate serum transaminase levels at any of the time points tested (Figures S2C and S2D), indicating that AAV treatment itself is not contributing to liver damage.

The body weight of *Lipa*^−/−^ mice did not differ from that of WT mice at any study time point (Figure S3A). Additionally, we saw a significant reduction in muscle mass in the *Lipa*^−/−^ mice, about 25% (Figures S3B and S3C). Due to an enlarged liver, *Lipa*^−/−^ mice present with a distended abdomen. We therefore performed open-field studies to determine if mobility was affected (Figures S3D–S3H). Fine movement (such as sniffing and grooming) and peripheral movement (movement at the periphery of the open-field area) were not affected in *Lipa*^−/−^ mice relative to WT, but center-cage-based ambulation and rearing were significantly decreased. All such measures were improved with treatment at all time points. Muscle atrophy may also contribute to ambulation differences in additional to abdominal distension in *Lipa*^−/−^ mice, and muscle weights were increased back to WT with P60 and P120 injection, while they only were marginally increased for P2 (Figures S3B and S3C).

### AAV vgs in organs result in *LIPA* expression and restored LAL enzyme activity

**Figure 2:**
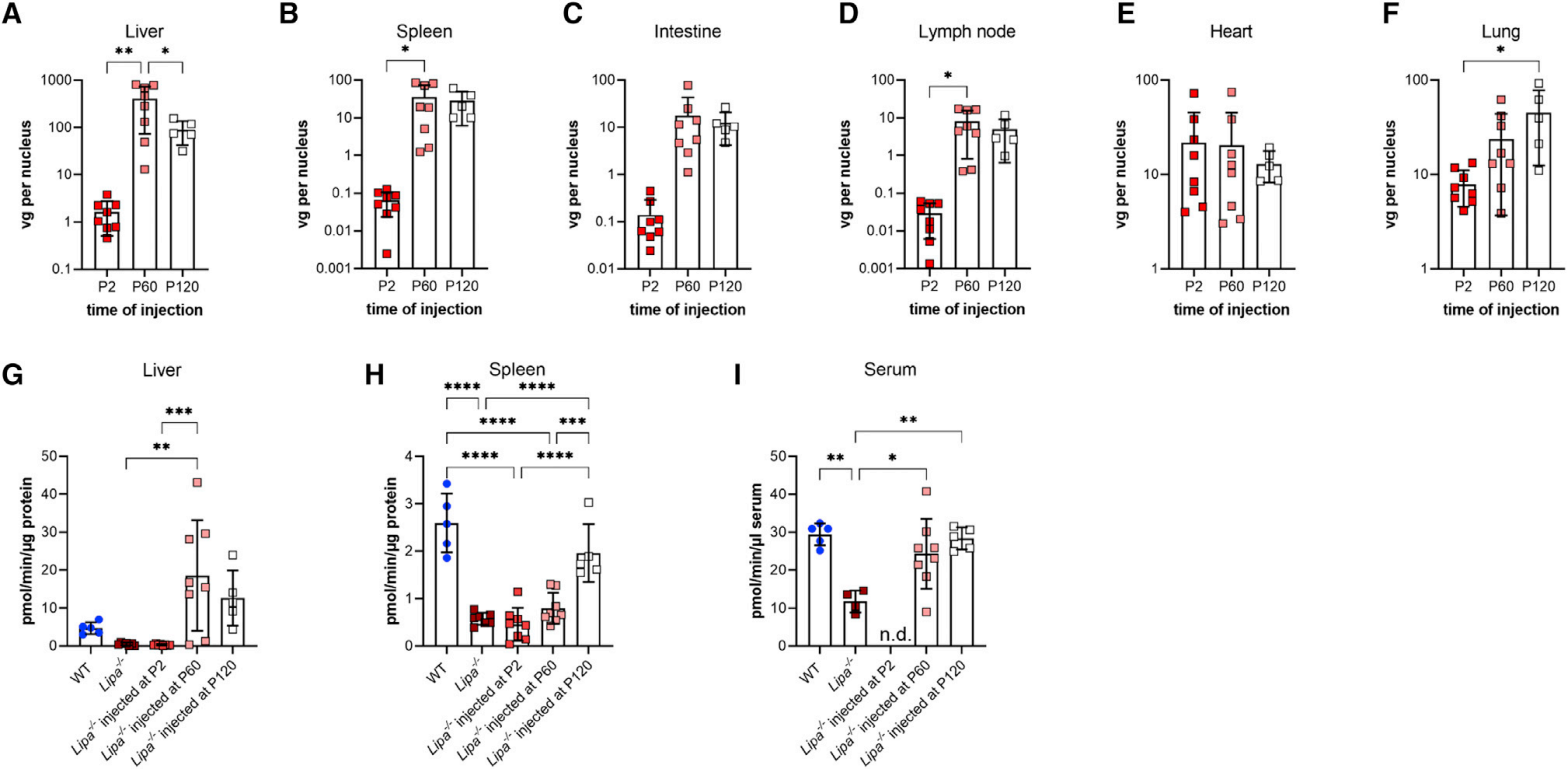
rscAAVrh74.miniCMV.*LIPA* biodistribution and increased lysosomal acid lipase enzyme activity (A–F) Biodistribution of AAV in various organs and tissues at 6 months of age following different treatment points. Vector genomes (vg) per nucleus were quantified using quantitative real-time PCR. (G–I) Lysosomal acid lipase enzyme activity in liver, spleen, and serum at 6 months of age following different treatment points. n.d., not determined. All data represented as mean ± SD (n = 5–8). Statistical significance was defined as p ≤ 0.05 (∗p ≤ 0.05, ∗∗p ≤ 0.01, ∗∗∗p ≤ 0.001, ∗∗∗∗p ≤ 0.0001) using one-way ANOVA with Tukey’s post-hoc test.

We next assessed biodistribution of AAV vgs in various organs and tissues from all injection time points using quantitative real-time PCR (Figures 2A–2F). At the 6-month endpoint, there was a difference in organ biodistribution between mice injected at P2 versus mice injected at P60 or P120; injections at P2 showed increased AAV vgs in the heart and lungs (21.82 ± 8.41 vg/nucleus and 7.87 ± 1.16 vg/nucleus, respectively; Table S1), while injection at P60 or P120 showed increased vgs in the liver (406.13 ± 117.59 vg/nucleus and 90.69 ± 21.48 vg/nucleus, respectively), with higher levels (20–50 vg/nucleus) also in kidney, lung, spleen, and thymus (Figures 2A–2F; Table S1). Comparison of kidneys and muscles from the left and right sides of the body plan suggested even AAV distribution. With injections at P2, vg levels were relatively stable over the course of the study in most organs, though there was a sharp decline in vgs between 4 and 6 months in the heart and liver (Figure S4A; Tables S1–S3). With injections at P60, vg levels were also stable when measured at 2 and 4 months post-injection (Figure S4B; Tables S1 and S2).

We next used quantitative reverse transcription (RT)-PCR to measure mRNA levels of the human *LIPA* transgene in treated *Lipa*^−/−^ mice compared with normal expression of endogenous mouse *Lipa* gene in WT tissues, normalized to 18S ribosomal RNA. In WT mice, *Lipa* is expressed at about the same levels in the liver, spleen, lymph nodes, kidneys, heart, lungs, thymus, and brain, with lower expression in intestines and muscles (Figure S5). At the 6-month study endpoint, there was a 3- and 15-fold increase in *LIPA* expression in the lung and heart, respectively, with injection at P2 (Figure S6). This was consistent with the finding of 7 and 20 vg/nucleus of AAV vgs in these organs at this time point. P2 injection achieved a near normal level of gene expression in liver, with the human *LIPA*/mouse WT *Lipa* ratio equal to 0.8. Interestingly there was only a 1.5-fold increase in liver in the same ratio for P60 or P120 injection at 6 months despite the fact that there was, on average, 90 or 400 vgs/nucleus in liver at this time point, respectively (Figure S6; Table S1). This suggests potential feedback inhibition (such as gene silencing or down-regulation) of AAV-mediated *LIPA* transgene expression with age or down-regulation of promoter activity.

We next measured LAL enzyme activity in liver, spleen, and serum from treated and control mice (Figures 2G–2I, S7, and S8). At the 6-month endpoint, overall LAL enzyme activity was reduced by 90% in *Lipa*^−/−^ liver relative to WT (Figure 2G). Liver enzyme activity did not significantly differ between the untreated *Lipa*^−/−^ mice and those treated at P2 at all examined time points (Figure 2G and S7A). When injected at P60 and P120, however, AAV treatment led to a significant increase in enzyme activity that exceeded WT activity by 4- and 2.5-fold, respectively. In spleen, overall LAL activity was reduced about 80% relative to WT at 6 months. Similar to liver, P2-injected spleens showed no improvement in activity at 6 months. Unlike liver, however, the P60 treatment time point in spleen also showed no improvement, while P120 did show an increase, though one that did not reach WT levels (Figure 2H and S7B). We also assayed serum LAL enzyme activity to determine whether exogenous LAL was being secreted from cells as a result of treatment in a manner that might be utilized in *trans* by other tissues (Figure 2I, S7, and S8). Overall, serum LAL activity in untreated *Lipa*^−/−^ mice was about one third that of the WT, and treatment at P60 and P120 elevated serum LAL enzyme activity back to WT levels (Figure 2I). The lack of reduction in serum LIPA activity to near baseline in *Lipa*^−/−^ mice may reflect complications in assaying LIPA activity in the presence of very high endogenous lipase activity in serum. Serum enzyme activity was not performed on P2-treated mice as there was not enough serum remaining after blood chemistry analysis.

### Triglyceride and cholesterol elevations are reduced in rscAAVrh74.miniCMV.*LIPA*-treated *Lipa*^−/−^mice

**Figure 3:**
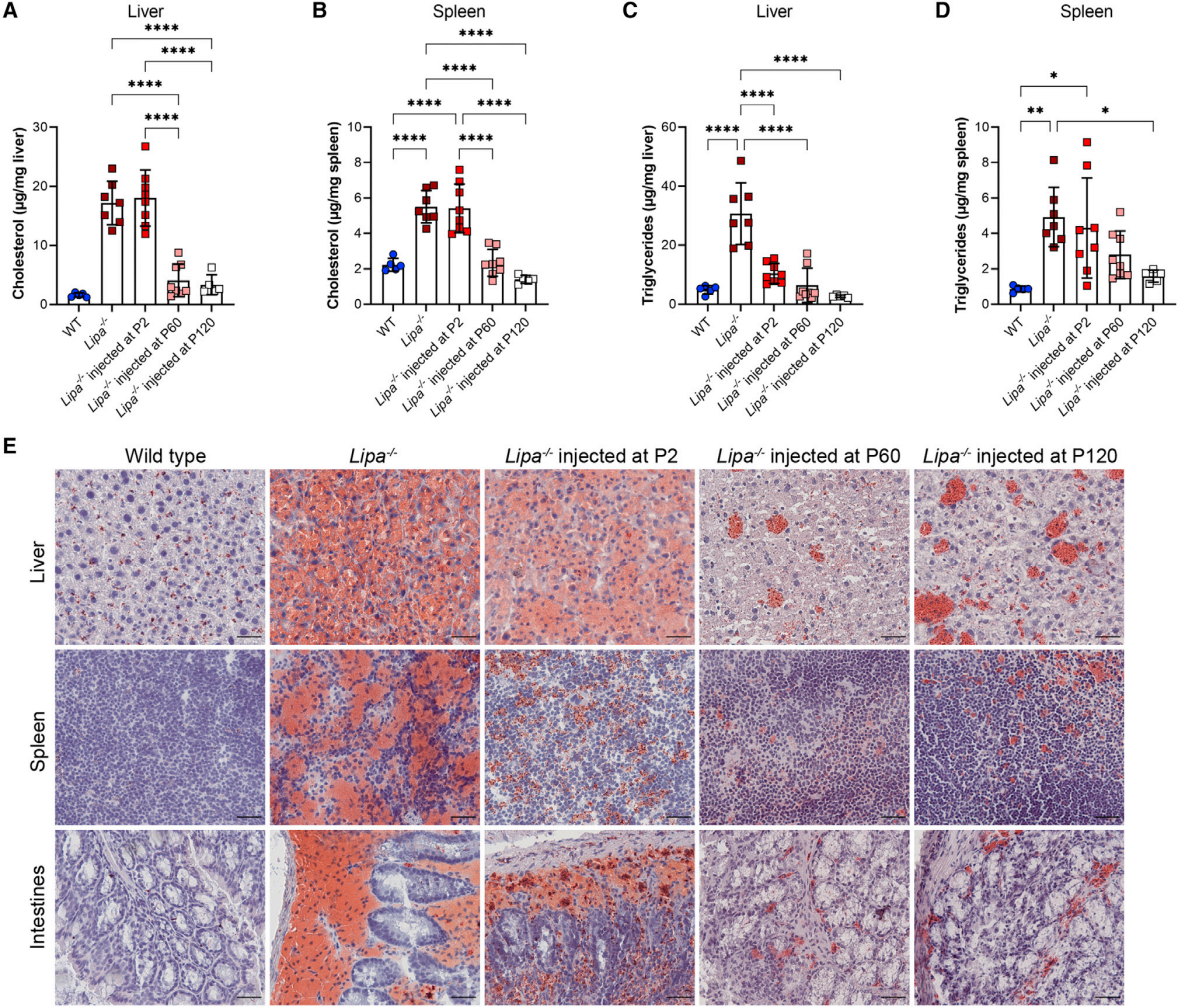
Cholesterol and triglyceride content is reduced with treatment at 6 months of age (A and B) Total cholesterol content in (A) liver and (B) spleen. (C and D) Triglyceride content in (C) liver and (D) spleen. (E) Oil red O (ORO) staining of liver, spleen, and intestines tissue sections at 6 months. Neutral lipids are stained red with ORO. Tissue sections were counter stained with hematoxylin (purple). Scale bar: 25 μm. All data represented as mean ± SD (n = 5–8). Statistical significance was defined as p ≤ 0.05 (∗p ≤ 0.05, ∗∗p ≤ 0.01, ∗∗∗p ≤ 0.001, ∗∗∗∗p ≤ 0.0001), using one-way ANOVA with Tukey’s post-hoc test.

When assayed at the 2-, 4-, and 6-month time points, total cholesterol in the liver of untreated *Lipa*^*−/−*^ mice was consistently elevated, on average 19.96 ± 3.97 μg/mg, a value approximately 12-fold higher than the WT (Figure 3A and S9A). Cholesterol content in the spleen increased more slowly, from 2.65 ± 0.66 μg/mg at 2 months up to 5.51 ± 0.91 μg/mg at 6 months (Figure S9B). Cholesterol content also increased with age in WT spleen (from 1.44 ± 0.70 μg/mg at 2 months to 2.23 ± 0.36 μg/mg at 6 months). Treatment at P2 significantly decreased cholesterol levels in the liver and spleen at 2 and 4 months post-injection. At 6 months, total cholesterol levels for P2 treatment reverted to untreated *Lipa*^−/−^ levels in both the liver and the spleen (Figures 3A and 3B), while cholesterol content remained reduced to near WT levels for P60 and P120 injection.

Triglyceride levels in the liver of untreated *Lipa*^−/−^ mice doubled between 2 and 4 months of age (14.04 ± 4.15 to 29.32 ± 3.70 μg/mg) and then remained constant at 6 months (30.66 ± 10.45 μg/mg) (Figure 3C and S9C). At 6 months, these values, on average, were elevated 6-fold compared with WT (average 4.70 ± 0.34 μg/mg). At all time points, treatment reduced liver triglycerides significantly, approaching or reaching WT-levels (Figure 3C and S9C). In the spleen, triglyceride content in *Lipa*^−/−^ mice increased more gradually between 2 and 6 months of age (2.27 ± 1.32 to 4.31 ± 2.82 μg/mg), and it was not until 6 months of age that *Lipa*^−/−^ spleen triglyceride content was significantly greater than WT. Unlike liver, treatment at P2 and P60 did not significantly alter triglyceride content in the spleen at the 2-, 4-, or 6-month endpoint (Figure 3D and S9D). Instead, only treatment at P120 showed a significant decrease.

Serum changes in *Lipa*^−/−^ mice included reduced total cholesterol, triglycerides, HDL cholesterol, and free fatty acids, along with elevated LDL cholesterol (Figure S10). P2 treatment did not significantly ameliorate any of these changed serum lipid levels at the 2-, 4-, or 6-month endpoint, but there were often trends toward improvement. By contrast, treatment at P60 and P120 offset reductions in total cholesterol, HDL cholesterol, and free fatty acids in *Lipa*^−/−^ mice, generating levels near those found in WT mice at the 6-month endpoint. Similarly, treatment partially corrected triglycerides and LDL cholesterol.

We used oil red O (ORO) staining of tissue sections to visualize neutral lipids in the liver, spleen, and intestine (Figure 3E). Compared with the WT, there were large accumulations of lipids stained with ORO in untreated *Lipa*^−/−^ liver, spleen, and intestine, with almost ubiquitous strong staining being present in the liver. With treatment at all time points, there was a decrease in overall ORO staining, but this was most pronounced for treatment at P60 and P120. After treatment, ORO staining appeared primarily as lipid islands within the tissue, with the more diffuse staining seen in untreated *Lipa*^−/−^ mice being absent from the remainder of the section.

### rscAAVrh74.miniCMV.*LIPA* treatment decreases liver inflammation and fibrosis

**Figure 4:**
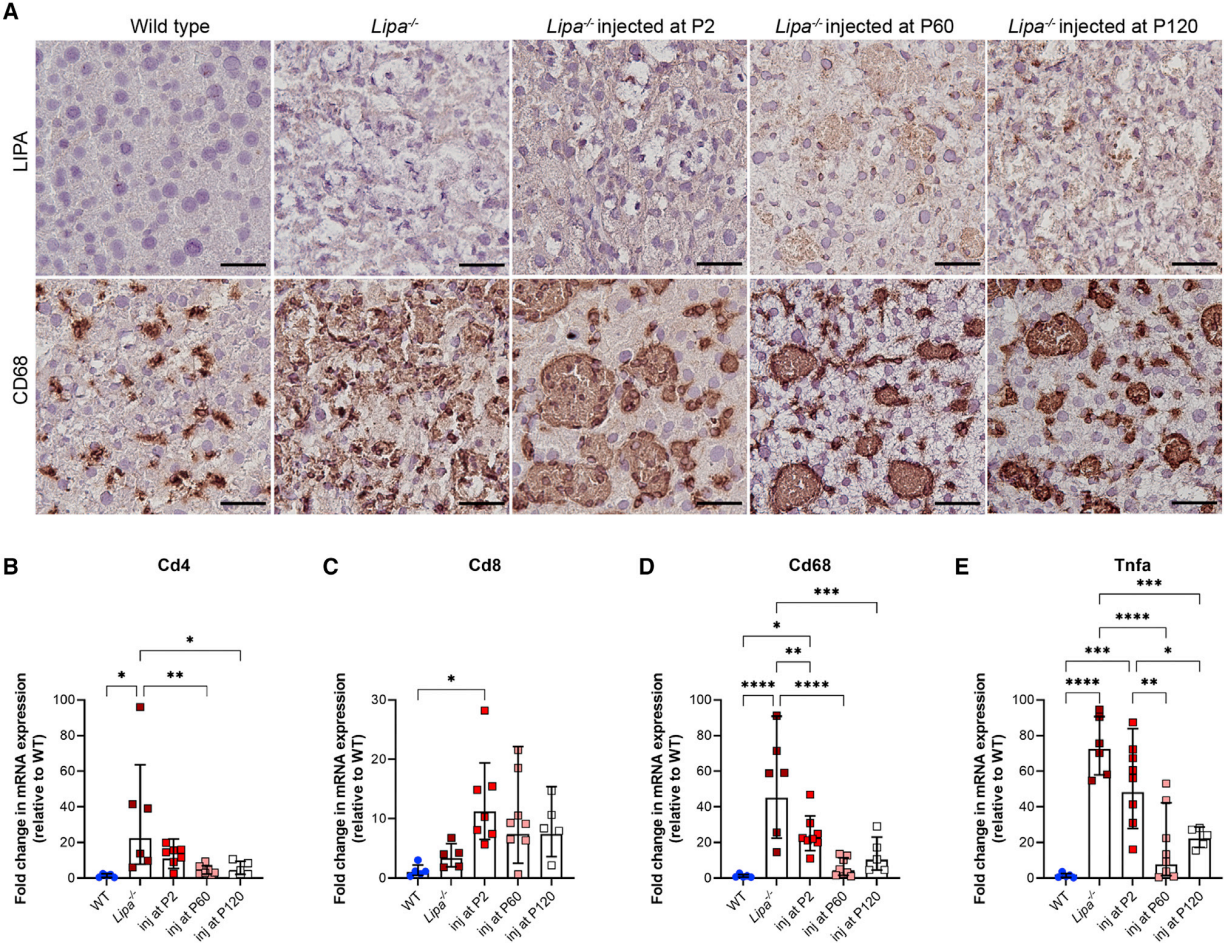
Inflammation is reduced in the liver after AAV treatment (A) LIPA immunostaining in treated livers (brown) (top panels). Macrophages are stained with anti-CD68 (brown) (bottom panels). Tissue sections were counter stained with hematoxylin (purple). Scale bar: 25 μm. (B–E) qRT-PCR expression of various markers of inflammation in the liver. All data represented as mean ± SD (n = 5–8). Statistical significance was defined as p ≤ 0.05 (∗p ≤ 0.05, ∗∗p ≤ 0.01, ∗∗∗p ≤ 0.001, ∗∗∗∗p ≤ 0.0001), using one-way ANOVA with Tukey’s post-hoc test.

We sought to determine LIPA protein expression and the extent of macrophage occupancy in livers by performing immunostaining at the 6-month endpoint (Figure 4A and S11). For P2-treated mice, immunohistochemistry (IHC) staining for LIPA revealed only very diffuse and weak staining in liver cells, with little to no punctate staining that would be expected for localization of protein to lysosomes. By contrast, *Lipa*^−/−^ mice treated at P60 and P120 showed increased punctate immunostaining for LIPA that was evident in the majority of cells throughout liver sections. Anti-CD68 staining, a marker used to detect macrophages and Kupffer cells, showed highly elevated staining in untreated *Lipa*^−/−^ mice. With treatment at all time points, there was a decrease in CD68^+^ macrophages, though staining remained within lipid islands present in the liver. Thus, LIPA and CD68 IHC staining supported the notion that P60 and P120 treatment led to high levels of LIPA expression in the liver and to a lowered macrophage occupancy. Immunofluorescence staining of liver sections at the 6-month endpoint also confirmed the expression of LIPA in hepatocytes and Kupffer cells (macrophages) through co-localization of LIPA with C-type lectin domain family 4 member F (CLEC4F), a Kupffer cell marker (Figure S11). LIPA expression was seen throughout the liver in hepatocytes and in discrete accumulations in Kupffer cells.

qRT-PCR analysis of liver tissue of select genes related to inflammation and immune response was performed to further assess the extent of response to therapy, including *tumor necrosis factor alpha* (*Tnfa*), a cytokine activated in macrophages as part of the inflammatory response, *Cd68*, a marker for macrophages (including Kupffer cells), and *Cd4* and *Cd8*, markers for helper and cytotoxic T cells (Figures 4B–4E). At the 6-month endpoint, expression of all assayed genes showed an increase in untreated *Lipa*^−/−^ mice compared with WT, suggesting increased inflammation. *Cd4*, *Cd68*, and *Tnfa* expression was reduced significantly in mice injected at P60 and P120 compared with untreated *Lipa*^−/−^ mice, as was *Cd68* expression in P2-treated mice. The decrease in *Cd68* expression was in agreement with decreased CD68 staining. *Cd8* expression, however, showed a trend toward increased expression at all times after treatment, consistent with a CD8 response to AAV infection, though such CD8 responses to AAV can be non-functional.^24^

We next looked at the extent of liver fibrosis in treated versus untreated *Lipa*^−/−^ mice. At the 6-month endpoint, treatment at P60 significantly decreased the amount of liver fibrosis as evident from Masson’s trichrome staining (Figure S12A). Treatment at P2 and P120 also decreased fibrosis, but the decrease was not as dramatic as treatment at P60. The reduction in fibrosis was also confirmed using qRT-PCR to determine the expression of 3 genes involved in fibrosis: *collagen 1A1* (*Col1a1*), a protein involved in matrix remodeling, *transforming growth factor-beta* 1 (*Tgfb1*), an inducer of fibrogenesis, and *tissue inhibitor of metalloproteinase-1* (*Timp1*), a protein produced by activated hepatic stellate cells and Kupffer cells that is involved in matrix remodeling during liver injury and repair (Figures S12B–S12D). *Col1a1* expression was elevated 100-fold in *Lipa*^−/−^ mice relative to WT, and these levels were significantly reduced with treatment at P60, consistent with reduced trichrome staining. *Timp1* was upregulated 300-fold in untreated *Lipa*^−/−^ mice compared with WT, and treatment at P60 and P120 significantly decreased expression. *Tgfb1* expression was elevated less than 10-fold in untreated *Lipa*^−/−^ mice relative to WT, an insignificant change, and this level was unchanged by treatment.

Finally, we performed hematoxylin and eosin (H&E) staining of treated livers to assess cellular morphology and evidence of hepatocellular carcinoma (HCC). Staining of treated *Lipa*^−/−^ mice, especially with treatment at P60, showed a reduction in steatosis (Figure S13). Staining of treated *Lipa*^−/−^ mouse livers showed no evidence of HCC at any time point (Figure S13).

### Lower doses of rscAAVrh74.miniCMV.*LIPA* still show therapeutic benefits

We initially treated at a dose of 8.4 x 10^13^ vg/kg to ensure saturation of the gene therapy treatment. Given the promising data from this dosage, we next sought to determine if lower doses of gene therapy would still prove efficacious. Since injection at P60 (mid-stage disease) provided the most positive results with the high dose, we repeated this injection protocol with 4.2 × 10^13^, 2.1 × 10^13^, 1.0 × 10^13^, 5.0 × 10^12^, and 1.0 × 10^12^ vg/kg of rscAAVrh74.miniCMV.*LIPA*, ultimately lowering the dose 80-fold relative to our starting dose. These mice were followed until 6 months of age (4 months post-injection). Three to six mice were included in each dose treatment group; however, only 2 mice that received a 5 × 10^12^ vg/kg dose survived to the 6-month endpoint.

**Figure 5:**
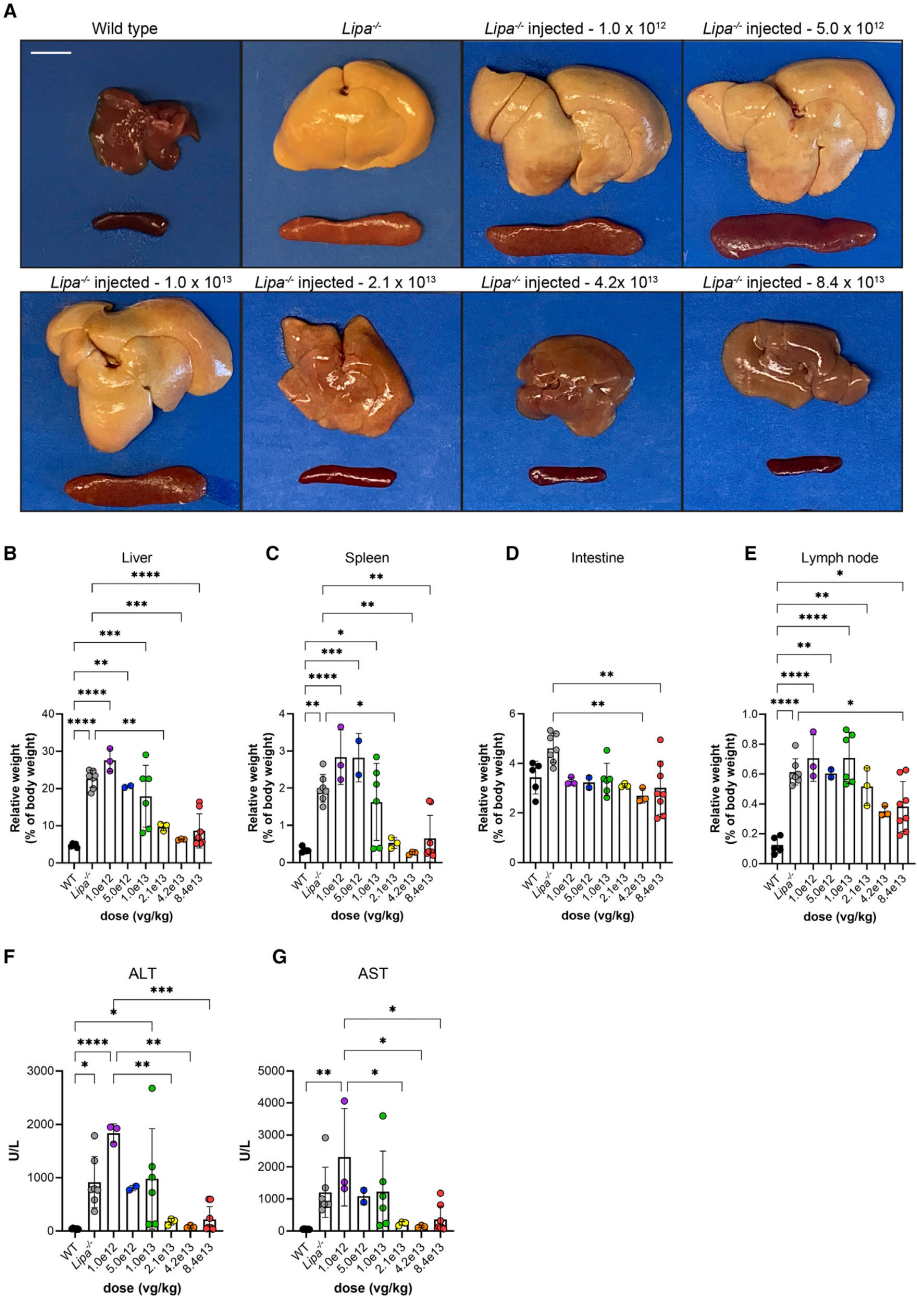
Lower doses of rscAAVrh74.miniCMV.LIPA still show therapeutic benefits (A) Gross pathology of the liver and spleen in the untreated WT and *Lipa*^−/−^ and treated *Lipa*^−/−^ at 6 months of age with different doses of rscAAVrh74.miniCMV.*LIPA*. Scale bar: 1 cm. (B–E) Relative weight of liver (B), spleen (C), intestines (D), and lymph node (E) after treatment with different doses at 6 months of age. (F and G) Serum ALT and AST levels with different doses at 6 months of age. All data represented as mean ± SD (n = 3–8). Statistical significance was defined as p ≤ 0.05 (∗p ≤ 0.05, ∗∗p ≤ 0.01, ∗∗∗p ≤ 0.001, ∗∗∗∗p ≤ 0.0001) using one-way ANOVA with Tukey’s post-hoc test.

Hepatosplenomegaly was significantly reduced with 2 of the new lower doses—4.2 × 10^13^ and 2.1 × 10^13^ vg/kg (Figure 5). Treatment at those 2 doses showed more normal liver and spleen appearance when compared with the remaining doses and untreated *Lipa*^−/−^ mice (Figure 5A), with the two highest doses (8.4 × 10^13^ and 4.2 × 10^13^ vg/kg) having slightly more effect than the lower doses. The relative weight for the liver and the spleen was significantly reduced compared with untreated *Lipa*^−/−^ mice with doses of 2.1 × 10^13^, 4.2 × 10^13^, and 8.4 × 10^13^ vg/kg (Figures 5B and 5C). Likewise, the two highest doses showed a reduction in the relative weight of the intestine (Figure 5D), while only the highest dose showed a reduction in the weight of the mesenteric lymph node (Figure 5E). Serum ALT and AST values were decreased with doses of 2.1 × 10^13^, 4.2 × 10^13^, and 8.4 × 10^13^ vg/kg, again with a trend toward higher levels at even lower doses (Figures 5F and 5G). Thus, a dose as low as 2.1 × 10^13^ vg/kg can significantly reduce hepatosplenomegaly and serum markers of liver damage.

**Figure 6:**
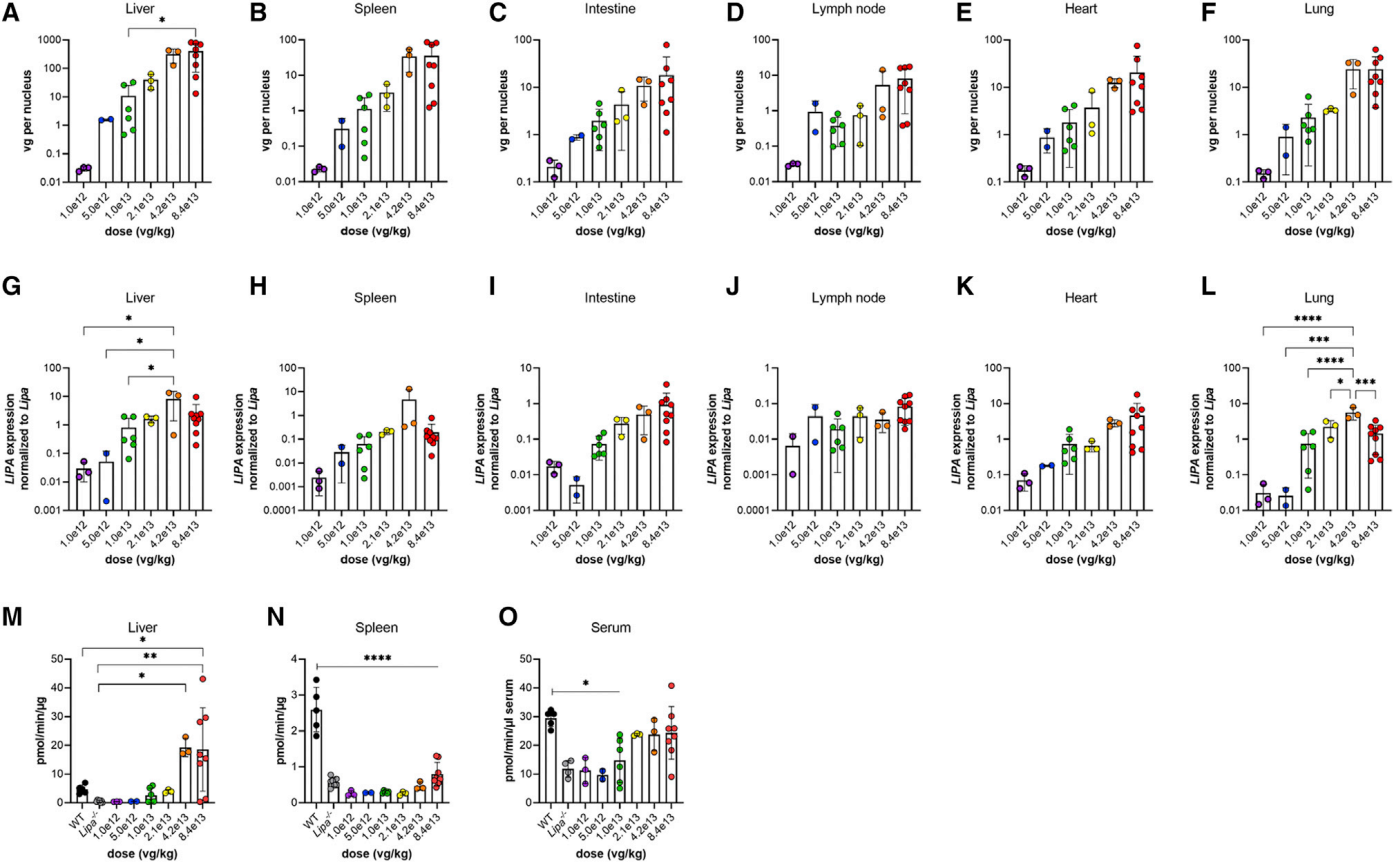
Reduced doses of rscAAVrh74.miniCMV.*LIPA* treatment results in restored *LIPA* expression and lysosomal acid lipase enzyme activity (A–F) Biodistribution of AAV decreases with decreasing dose in the liver, spleen, intestine, lymph node, heart, and lung. (G–L) *LIPA* expression also decreases with dose in the liver, spleen, intestine, lymph node, heart, and lung. (M–O) Lysosomal acid lipase activity in the liver, spleen, and serum. All data represented as mean ± SD (n = 2–8). Statistical significance was defined as p ≤ 0.05 (∗p ≤ 0.05, ∗∗p ≤ 0.01, ∗∗∗p ≤ 0.001, ∗∗∗∗p ≤ 0.0001), using one-way ANOVA with Tukey’s post-hoc test.

Biodistribution of AAV was examined in all organs and tissues for the dosing experiment (Figures 6A–6F; Table S4). There was a 2-fold decrease in AAV vgs/nucleus between doses 8.4 × 10^13^ versus 4.2 × 10^13^ and 2.1 × 10^13^ versus 1.05 × 10^13^ vg/kg, while there was a steeper than 2-fold decrease in AAV vgs/nucleus between doses of 4.2 × 10^13^ and 2.1 × 10^13^ vg/kg in all organs. Interestingly, elevated AAV biodistribution translated to less expected human *LIPA* transcript expression (Figures 6G–6L); in the liver, at the highest dose (8.4 × 10^13^ vg/kg), there was only about a 2-fold increase in *LIPA* expression, normalized to endogenous mouse WT *Lipa*, despite there being more than 200 vg/nucleus. Similar disparities, though less extreme, were seen in spleen, intestine, lymph node, heart, and lung. At a dose of 1.0 × 10^13^ vg/kg, however, *LIPA* transcript levels were still restored to WT levels in the liver (Figure 6G), resulting in WT levels of enzyme activity in the liver (Figure 6M). Doses of 2 × 10^13^ vg/kg and higher led to normal or supranormal enzyme activity levels in the liver and to serum levels that were not significantly different from WT (Figure 6M). Enzyme activity levels in spleen, by contrast, were not normalized at any dose (Figure 6N), possibly due to the very low *LIPA* gene expression levels seen (Figure 6H).

**Figure 7:**
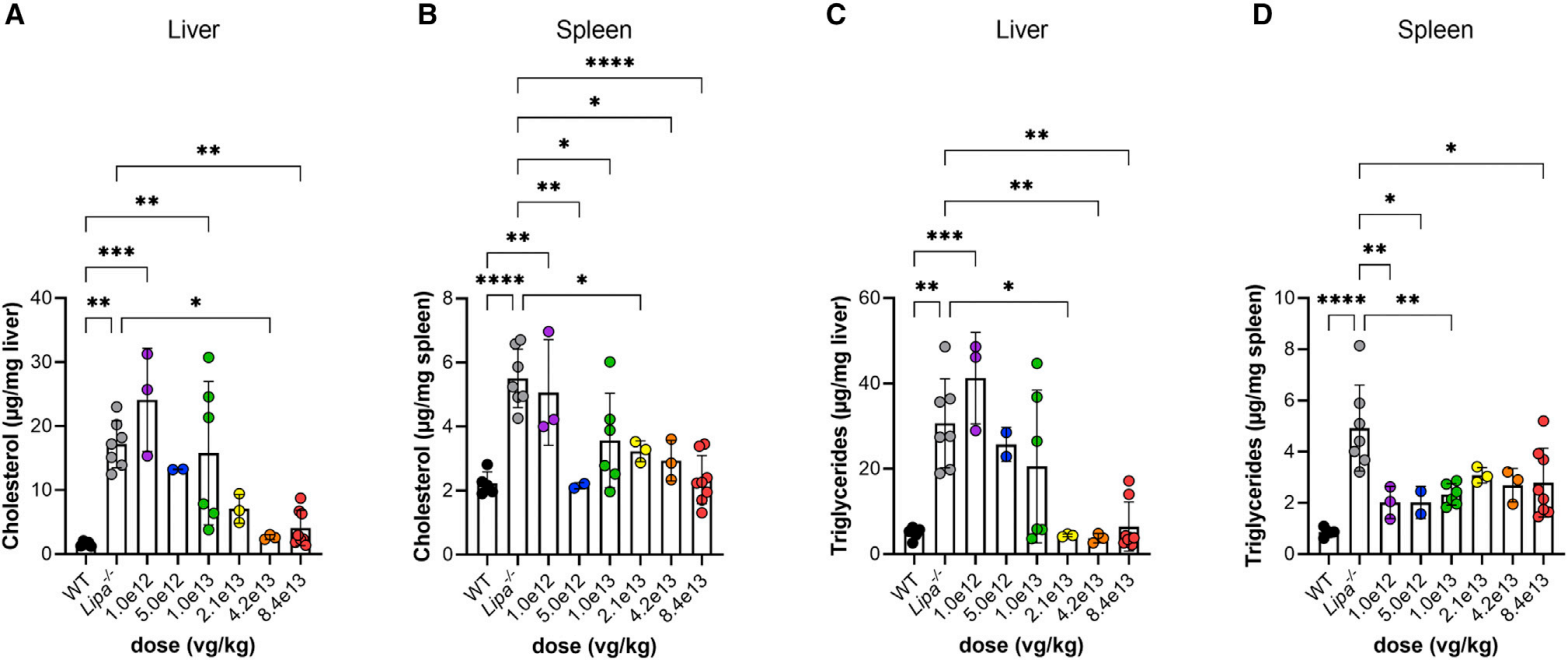
Definition of AAV dose required to lower triglyceride and cholesterol organ content (A) Cholesterol content in the liver. (B) Cholesterol content in the spleen. (C) Triglyceride content in the liver. (D) Triglyceride content in the spleen. All data represented as mean ± SD (n = 2–8). Statistical significance was defined as p ≤ 0.05 (∗p ≤ 0.05, ∗∗p ≤ 0.01, ∗∗∗p ≤ 0.001, ∗∗∗∗p ≤ 0.0001) using one-way ANOVA with Tukey’s post-hoc test.

The three highest doses also significantly reduced cholesterol (Figure 7A) and triglyceride (Figure 7C) content in liver relative to untreated *Lipa*^−/−^ mice. Spleen cholesterol (Figure 7B) was reduced to WT, or nearly equivalent to WT, levels with the three highest doses, and spleen triglyceride levels (Figure 7D) were reduced as well. Thus, reductions in liver and spleen lipid content can be achieved in *Lipa*^*−/−*^ mice with a dose of rscAAVrh74.miniCMV.*LIPA* as low as 2.1 × 10^13^ vg/kg. A similar trend in reduced expression for markers of inflammation and fibrosis was also seen (Figures S14 and S15).

## Discussion

Intravenous injection of rscAAVrh74.miniCMV.*LIPA* largely corrected many of the phenotypes of LAL-D in a *Lipa*^−/−^ mouse model, including hepatosplenomegaly, elevated serum transaminases, reduced LAL activity, and cholesterol and triglyceride accumulation in organs, demonstrating that AAV gene therapy may be a viable approach to treating LAL-D. We observed that treatment at later disease stages (P60 and P120) showed greater reduction in disease symptoms than injection at an early stage (P2) and that this corresponded with a decrease in AAV vgs in the liver with P2 injection. This is presumably due to the rapid proliferation of hepatocytes that occurs in mice during their first post-natal month and to the fact that the AAV genome largely remains episomal and therefore cannot be efficiently passed onto the progeny of dividing cells. In adult mice, hepatocytes proliferate slowly, about once every 180–400 days.^25^^,^^26^ This slow growth would allow for stable expression of AAV transcripts and prolonged therapeutic effects of gene therapy as seen with treatment at P60 and P120. We observed less vgs in the liver with treatment at P60 versus P120. Though liver fibrosis and cirrhosis may not have an effect on AAV transduction,^27^ pre-existing liver injury or increased steatosis may. In neonatal mice, the liver increases in mass more than 30-fold, with 1–2 doublings occurring by the second week of life.^25^^,^^26^ The decrease in AAV vgs in the liver over time in P2-treated mice is consistent with other studies where injection of neonatal mice with AAV2/8 vectors shows reduced EGFP expression over time due to vector dilution.^28^^,^^29^ Our results show that treatment at P2 is sustained at high enough levels to inhibit disease phenotypes up to 4 months post-injection, as was also seen for AAV-mediated gene therapy of mucopolysaccharidosis type VII (MPS VII), a lysosomal storage disease,^30^ but then waned in efficacy. Overall, these results suggest AAV treatment may be very effective in patients with CESD, where treatment could occur after the period of robust liver growth, but may be more limited in infants with WD, where liver growth has largely yet to occur. The decrease in AAV vgs over time with neonatal injection in mice suggests that re-administration of gene therapy may be necessary for successful treatment in humans for infantile-onset diseases, such as WD, or that treatment with a therapy that integrates the gene into the genome, such as lentivirus, may be required.

In this study, we used a minimal CMV promoter to drive expression of the human *LIPA* transgene. This promoter was chosen as it is a constitutive strong promoter that can provide robust expression of *LIPA* to multiple tissue types. LAL-D is a multi-system disorder, affecting liver, spleen, intestines, lungs, brain, and blood, as well as bone.^31^^,^^32^ We sought to get *LIPA* expression throughout the body to get maximal *trans* effects from overexpression, as extracellular LAL can be taken up by other cells though mannose 6-phosphate receptor-mediated endocytosis,^33^ much as occurs with enzyme replacement therapy. Though we saw high levels of AAV vgs in many organs, including the liver, we often did not detect corresponding increases in gene expression. Low transcript levels may be due to transcriptional regulation of the *LIPA* gene itself, though the exact mechanism is unknown.^2^ Another possibility for low *LIPA* transcript level is inactivation of the CMV promoter through methylation.^34^^,^^35^ To address the problem of CMV promoter inactivation, we could use a different promoter to drive expression of *LIPA* that may be less affected by methylation or use a liver-specific promoter. Use of a liver-specific promoter to restrict transgene expression to hepatocytes has been shown to induce antigen-specific tolerance, thereby enhancing the safety profile.^36^ While liver transplantation studies in patients with LAL-D showed some clinical efficacy, suggesting that liver is the primary organ in need of treatment, liver-specific deletion of LIPA in mice fails to manifest many aspects of LAL-D,^37^ which suggests that constitutive organ expression may be needed for maximal therapeutic benefit. While we did observe increased LAL enzyme activity in the liver at P60 and P120, this did not completely correct disease phenotypes in *Lipa*^−/−^ mice. This may suggest that the enzyme is not being efficiently transported to dividing cells throughout the body or efficiently targeted in those cells to the lysosome. Further studies will be needed to better understand these issues.

We used rAAVrh74 for this study, a serotype recently shown to be safe and effective in patients with Duchenne muscular dystrophy.^38^ Seroprevalence of AAVrh74 in humans is low, which potentially may allow for its use in more patients.^39^ This serotype has also been used extensively in clinical trials, and AAV been shown to be safe when administered at high doses (2.0 × 10^14^ vg/kg).^38^^,^^40^ While rAAVrh74 shows excellent transduction in skeletal muscle and heart when given intravenously, it is still largely a liver-targeted AAV serotype,^41^ making it appropriate for treatment of LAL-D.

We investigated efficacy of 6 different doses, 8.4 × 10^13^, 4.2 × 10^13^, 2.1 × 10^13^, 1.0 × 10^13^, 5.0 × 10^12^, and 1.0 × 10^12^ vg/kg, injected at P60. The three highest doses improved the gross pathology of the liver and spleen, hepatosplenomegaly, and serum markers of liver damage. Additionally, LAL enzyme activity was restored to WT levels in the liver, and triglyceride and cholesterol content in organs was significantly reduced. All of these studies suggest that a dose as low as 2.1 × 10^13^ vg/kg may be an effective dose for therapy. This dose is in the range currently used in clinical and pre-clinical treatment of Niemann-Pick disease and various lysosomal storage disorders.^42^, ^43^, ^44^, ^45^, ^46^, ^47^ To further decrease the suggested dose, we could use serotypes that have increased tropism to the liver; AAV-DJ, for example, is an engineered strain created by capsid shuffling that has been shown to be highly specific to the liver.^48^

The current standard of treatment for LAL-D is weekly or bi-weekly infusions of enzyme replacement therapy (ERT) of sebelipase alfa (Kanuma), a recombinant form of the human LAL enzyme.^4^ Though treatment with sebelipase alfa has been shown to improve symptoms of LAL-D, ERT is time consuming, extremely expensive, and requires a continual bi-weekly treatment regimen. Additionally, expression into multiple tissue types through AAV-mediated gene therapy can help get *LIPA* expression into organs where ERT would be less effective, for example in the brain. This study has shown that one-time gene-replacement therapy for LAL-D is a feasible treatment alternative. For WD (early onset, severe form), gene-replacement therapy could be used in infants at the time of diagnosis, but likely would require re-administration later in life, or be used in combination with ERT.^49^ For CESD (the less severe, later onset form), our data suggest that a single gene therapy may be sufficient for effective treatment in adults and possibly teens.

## Materials and methods

### Mice

All mouse experiments were performed using protocols approved by the Institutional Animal Care and Use Committee at the Abigail Wexner Research Institute at Nationwide Children’s Hospital (Columbus, OH, USA). WT FVB/NJ mice were purchased from Jackson Laboratories (Bar Harbor, ME, USA). *Lipa*^−/−^ mice, bred in the FVB/NJ strain background, were a generous gift from Hong Du (Indiana University School of Medicine, Indianapolis, IN, USA). *Lipa*^−/−^ mice were bred from female heterozygotes and male homozygotes. Genotyping by PCR was performed to determine homozygous *Lipa*^−/−^ mice. DNA was extracted following the Dilution and Storage protocol from the Phire Tissue Direct PCR Master Mix kit (Thermo Fisher Scientific, Waltham, MA, USA). PCR was performed with Phire Tissue Direct Master Mix using 10 pmol of each primer in a 20-μL reaction as per manufacturer’s protocol. Primers (Integrated DNA Technologies, Coralville, IA, USA) used for genotyping are listed in Table S5. Littermate controls were used for all experiments and were age and sex matched. All animals were housed in a 12-h light-dark cycle with *ad libitum* access to food and water. All animals were fed a diet of standard chow.

### Cloning and production of AAV vector

The human *LIPA* gene coding sequence (GenBank: NM_000235.4) was synthesized by Twist Biosciences (South San Francisco, CA, USA) and cloned into the scAAV vector pTRS (obtained from Douglas McCarty, Nationwide Children’s Hospital, Columbus, OH, USA) under control of the minimal CMV (miniCMV) promoter (pTRS.miniCMV.hLIPA). The complete sequence of the therapeutic cassette is provided in Table S6. Sanger sequencing was performed to confirm the sequence of the plasmid and integrity of the inverted terminal repeats (Genewiz, South Plainfield, NJ, USA). Packaged AAV vector rscAAVrh74.miniCMV.*LIPA* was made by a triple-transfection method in HEK293 cells and purified using iodixanol density centrifugation and anion exchange chromatography by Andelyn Biosciences (Columbus, OH, USA) using previously described methods.^50^^,^^51^ AAV titers were measured using digital droplet PCR.

### rscAAVrh74.miniCMV.LIPA treatment of mice

One- to two-day-old FVB/NJ and *Lipa*^−/−^ mice were injected with rscAAVrh74.miniCMV.*LIPA* via the superficial temporal facial vein injection using an injection volume of 30 μL. Two- and -four-month-old FVB/NJ and *Lipa*^−/−^ mice were injected via the lateral tail vein using an injection volume of 200 μL. Each treatment group had 3–8 mice each. After 2, 4, and 6 months of treatment, mice were necropsied, and organs (liver, spleen, kidneys, intestine, mesenteric lymph node, heart, lung, thymus, brain) and muscles (left and right gastrocnemius and quadriceps) were harvested for biodistribution and gene expression. Harvested non-muscle organs were weighed and then immersed in OCT before being frozen in dry-ice-cooled isopentane. Muscles were weighed and then snap frozen in liquid-nitrogen-cooled isopentane.

### Open-field studies

Open-field tests to determine fine, ambulatory, center, peripheral, and rearing movement events were performed as previously described.^52^

### qPCR and qRT-PCR

Taqman qPCR was used to quantify AAV vgs and gene expression, much as previously described.^53^ Primer/probe sets were obtained from Integrated DNA Technologies (Coralville, IA, USA), or Thermo Fisher Scientific (Waltham, MA, USA). Sequences are listed in Table S5.

### Blood serum analysis

Blood was collected from mice at various time points via the submandibular vein. Blood was incubated at room temperature for 1 h, then centrifuged at 3,500 RPM for 10 min. Serum was separated from the blood, and both fractions were stored at −80°C until further analysis. Serum ALT, AST, HDL cholesterol, LDL cholesterol, total cholesterol, triglycerides, and free fatty acids measurements were performed by Antech GLP (Morrisville, NC, USA).

### Lipid analysis

Total lipids were extracted from snap-frozen tissues using the Lipid Extraction Kit (Chloroform Free) (Abcam, Waltham, MA, USA) as per manufacturer’s protocol. Triglycerides were measured using the Infinity Triglycerides Reagent (Thermo Fisher Scientific, Waltham, MA, USA), and total cholesterol was measured using the Infinity Cholesterol Reagent (Thermo Fisher Scientific, Waltham, MA, USA). Lipid concentrations were determined against a standard curve of triglycerides or cholesterol standards (Pointe Scientific, Canton, MI, USA). Measurements were performed in triplicate, and absorbance values at 500 nm were measured on a Synergy 2 plate reader (BioTek Instruments, Winooski, VT, USA).

### Histological analysis

Ten-μm frozen tissue sections were prepared from liver, spleen, and intestine. For ORO staining, tissue section slides were fixed in 10% neutral buffered formalin (Thermo Fisher Scientific, Waltham, MA, USA) for 2 min, rinsed in tap water, then immersed in propylene glycol for 2 min. Slides were then incubated in 0.5% ORO Solution (Sigma Aldrich, St. Louis, MO, USA) for 10 min, then differentiated in 85% propylene glycol for 1 min. Slides were counterstained with Mayer’s Modified hematoxylin (Thermo Fisher Scientific, Waltham, MA, USA), then mounted in Aquatex mounting medium (EMD Millipore, Burlington, MA, USA) with a glass coverslip. For H&E staining, sections were fixed in 10% neutral buffered formalin for 2 min, rinsed in tap water, stained with Gill’s Hematoxylin (Thermo Fisher Scientific, Waltham, MA, USA) for 1 min, washed in warm tap water, and then immersed in Shandon bluing reagent for 30 s (Thermo Fisher Scientific, Waltham, MA, USA). Slides were then washed in warm tap water, stained with Eosin-Y (Thermo Fisher Scientific, Waltham, MA, USA) for 30 s before dehydration in a series of ethanol, and cleared in xylene. Slides were mounted in cytoseal XYL mounting media (Thermo Fisher Scientific, Waltham, MA, USA) with a glass coverslip. Trichrome staining was performed using the Trichrome Stain (Masson) Kit (Sigma Aldrich, St. Louis, MO, USA) as per manufacturer’s protocol.

### IHC and immunofluorescent (IF) staining

Ten-μm frozen tissue sections were prepared from liver samples. Slides were fixed in acetone for 10 min at −20°C, allowed to air dry to evaporate excess acetone, and washed 2× in PBS. For IHC, slides were incubated in BLOXALL Endogenous Blocking Solution (Vector Laboratories, Burlingame, CA, USA) for 10 min, washed 2× in PBS, then blocked in 2.5% normal serum for 30 min. Slides were incubated overnight at 4°C with antibodies to LIPA (1:1000; HPA057052, Sigma Aldrich, St. Louis, MO, USA) or CD68 (1:500; MCA1957GA, Bio-Rad, Hercules, CA, USA). Following incubation, slides were washed 2× in PBS and incubated with ImmPRESS HRP Reagent (Vector Laboratories, Burlingame, CA, USA) for 30 min. Slides were then washed 2× in PBS and stained with ImmPACT DAB staining solution (Vector Laboratories, Burlingame, CA, USA) for 5 min. They were then counterstained with Hematoxylin QS (Vector Laboratories, Burlingame, CA, USA), dehydrated, differentiated, and mounted in Cytoseal XYL (Thermo Fisher Scientific, Waltham, MA, USA) with a glass coverslip. For IF, slides were blocked in 10% donkey serum for 1 h at room temperature, then incubated overnight at 4°C with antibodies to LIPA (1:100; HPA057052, Sigma Aldrich, St. Louis, MO, USA). Following incubation, slides were washed 3× in PBS and incubated with secondary antibody (1:1,000 donkey anti-rabbit Alexa Fluor 555, Thermo Fisher Scientific, Waltham, MA, USA) and CLEC4F-AF647 (1:200; 156803, Biolegend, San Diego, CA, USA) at room temperature for 1 h. Slides were then washed 3× in PBS and mounted in Prolong Gold with DAPI (Thermo Fisher Scientific, Waltham, MA) with a glass coverslip.

### Microscopy imaging

ORO, H&E, trichrome, and IHC staining were imaged on a Zeiss Axioskop 40 microscope at 40× magnification. Images were taken using an Axiocam 305 color camera using Zen blue v.3.4 software (Zeiss, Jena, Germany). Fluorescent imaging was performed on a Nikon ECLIPSE Ti2-E microscope at 60× magnification using NIS Elements software (Nikon, Tokyo, Japan). Images were processed using Fiji.^54^

### LAL enzyme assay

Frozen liver and spleen samples were homogenized in LAL tissue extraction buffer (0.1 M sodium phosphate [pH 6.8], 1 mM EDTA, 0.02% sodium azide, 10 mM DTT, 0.5% NP-40). Protein concentrations were determined with the bicinchoninic acid assay (Pierce, Rockford, IL, USA), using BSA as the standard. LAL activity was determined using 4-methylumbelliferyl palmitate (4-MUP; Gold Biotechnology, St. Louis, MO, USA) as the substrate, as previously described.^55^^,^^56^ Briefly, 1 μg of protein or 1 μL serum was added to 0.345 mM substrate solution (0.345 mM 4-MUP, 90.9 mM sodium acetate [pH 4.0], 1% [v/v] Triton X-100 and 0.0325% [w/v] cardiolipin), and enzymatic reactions were performed in triplicate in the presence or absence of the LAL inhibitor Lalistat2 (Sigma Aldrich, St. Louis, MO, USA). Reactions were incubated at 37°C for 3 h in the dark. Reactions were terminated by adding 200 μL of 150 mM EDTA (pH 11.5). A standard curve was prepared ranging from 0–33.3 μM 4-methylumbelliferone (4-MU; Gold Biotechnology, St. Louis, MO, USA). Fluorescence was measured on a SpectraMax M2 plate reader (Molecular Devices, San Jose, CA, USA) using a 355-nm excitation filter and a 460-nm emission filter. LAL activity (pmol/min/μg) was calculated by subtracting the enzymatic activity of the inhibited reaction from that of the uninhibited reaction.

### Statistical analysis

Determination of significance between more than 2 groups was made using one- or two-way analysis of variance (ANOVA) with post-hoc Tukey’s honestly significant difference (HSD) test. p *≤*0.05 was considered significant. All unmarked comparisons reflect non-significant (p > 0.05) differences. Statistical tests were performed using GraphPad Prism software (v.9.0.0. GraphPad Software, San Diego, CA, USA).

## Data Availability

All data and supporting materials are available within the article and supplemental information.

## Supplemental information

Document S1. Figures S1–S15 and Tables S1–S6

Document S2. Article plus supplemental information
