# Supplementary material for: Therapeutic efficacy of rscAAVrh74.miniCMV.LIPA gene therapy in a mouse model of lysosomal acid lipase deficiency
Source: Mol Ther Methods Clin Dev. 2022 Aug 4;26:413–26. doi: 10.1016/j.omtm.2022.08.001 (PMC9403906; doi:10.1016/j.omtm.2022.08.001)
Supplement: Document S1. Figures S1–S15 and Tables S1–S6 [file mmc1.pdf]

OMTM, Volume *26*

Supplemental information

Therapeutic efficacy of rscAAVrh74.miniCMV.*LIPA* gene therapy in a mouse model of lysosomal acid lipase deficiency

Patricia Lam, Anna Ashbrook, Deborah A. Zygmunt, Cong Yan, Hong Du, and Paul T. Martin

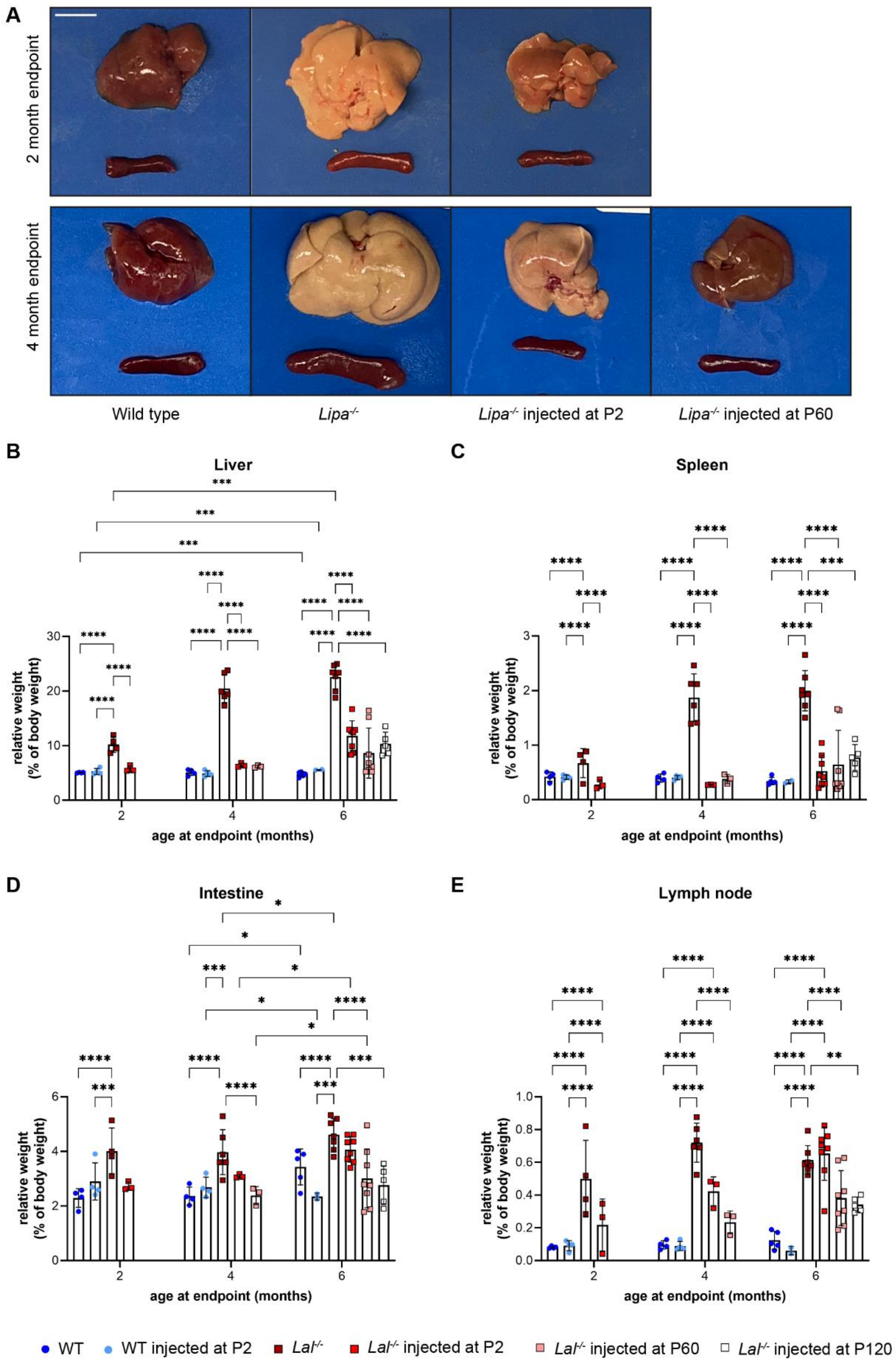

Figure S1. Organ morphology and weight at study endpoints.

(A) Gross morphology of liver and spleen at 2 months and 4 months of age. Scale bar = 1 cm. **(B-D)** Relative weights of the liver, spleen, intestine, and lymph node at 2, 4, and 6 months of age. All data represented as mean ± SD (n=3-8). Statistical significance was defined as p≤0.05 (*p≤0.05, **p≤0.01, ***p≤0.001, ****p≤0.0001), using two-way ANOVA with Tukey's post-hoc test.

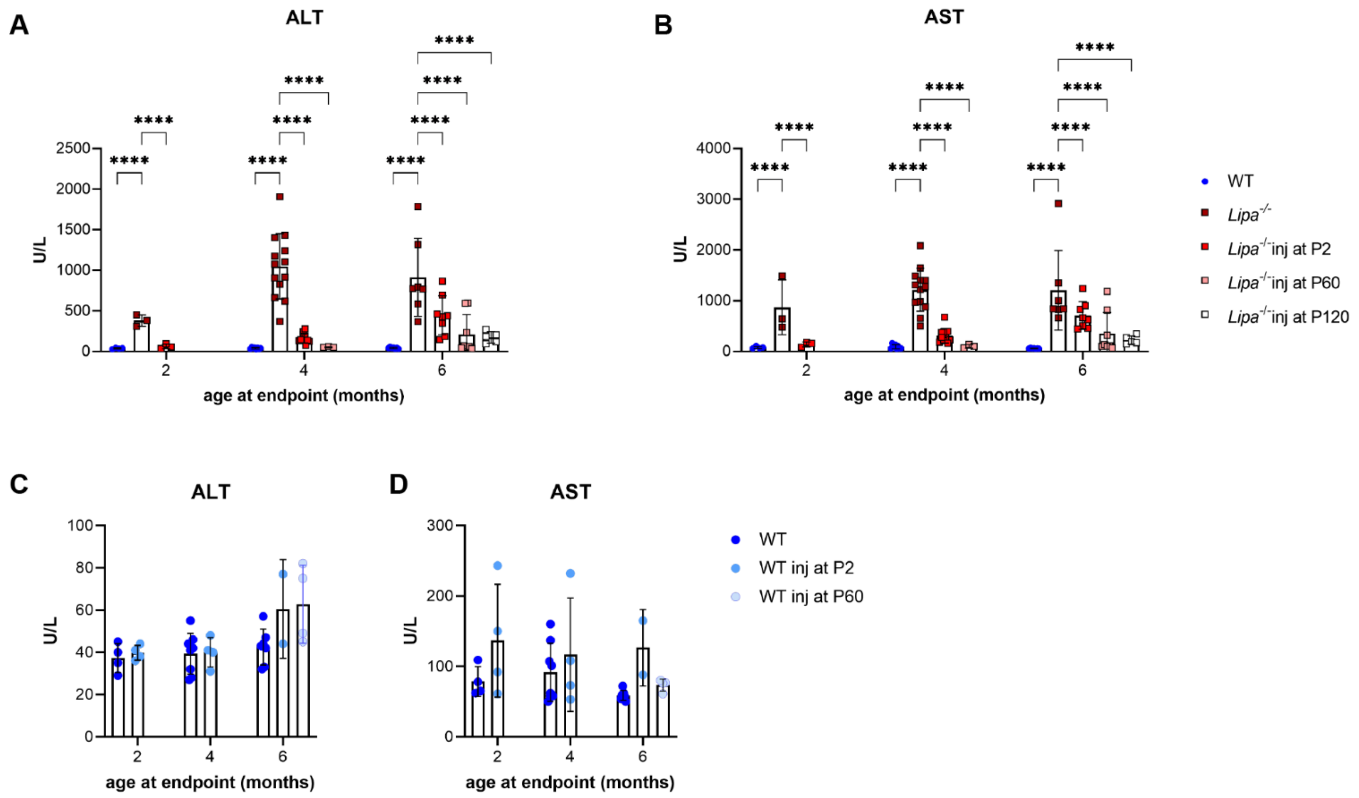

Figure S2. Serum transaminase levels at 2, 4, and 6 months of age.

(A) Serum ALT and **(B)** AST at 2, 4, 6 month endpoints of treated *Lipa*$^{-/-}$ mice. **(C)** Serum ALT and **(D)** AST at 2, 4, and 6 month endpoints of treated WT mice. All data represented as mean ± SD (n=2-8). Statistical significance was defined as p≤0.05 (****p≤0.0001) using two-way ANOVA with Tukey's post-hoc test.

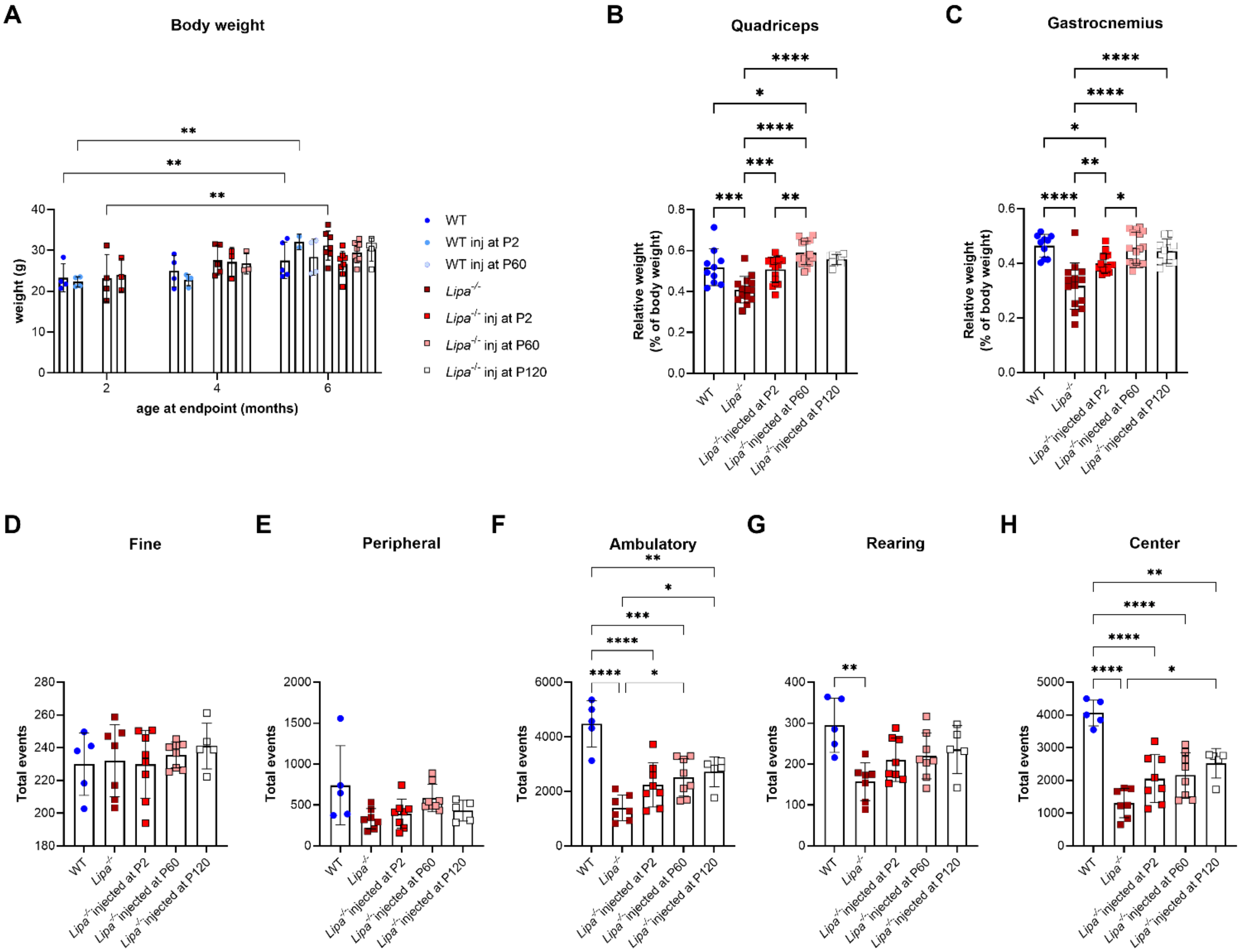

Figure S3. Muscle atrophy may contribute to ambulation differences in *Lipa*$^{-/-}$ mice.

(A) Body weight of mice at 2, 4, 6 months. **(B-C)** Relative weight of gastrocnemius muscle and quadricep muscle at 6 months. **(D-H)** Open field analysis at 6 months. All data represented as mean ± SD (n=5-8). Statistical significance was defined as p≤0.05 (*p≤0.05, **p≤0.01, ***p≤0.001, ****p≤0.0001), using two-way ANOVA (A) one-way ANOVA (B-H) with Tukey’s post-hoc test.

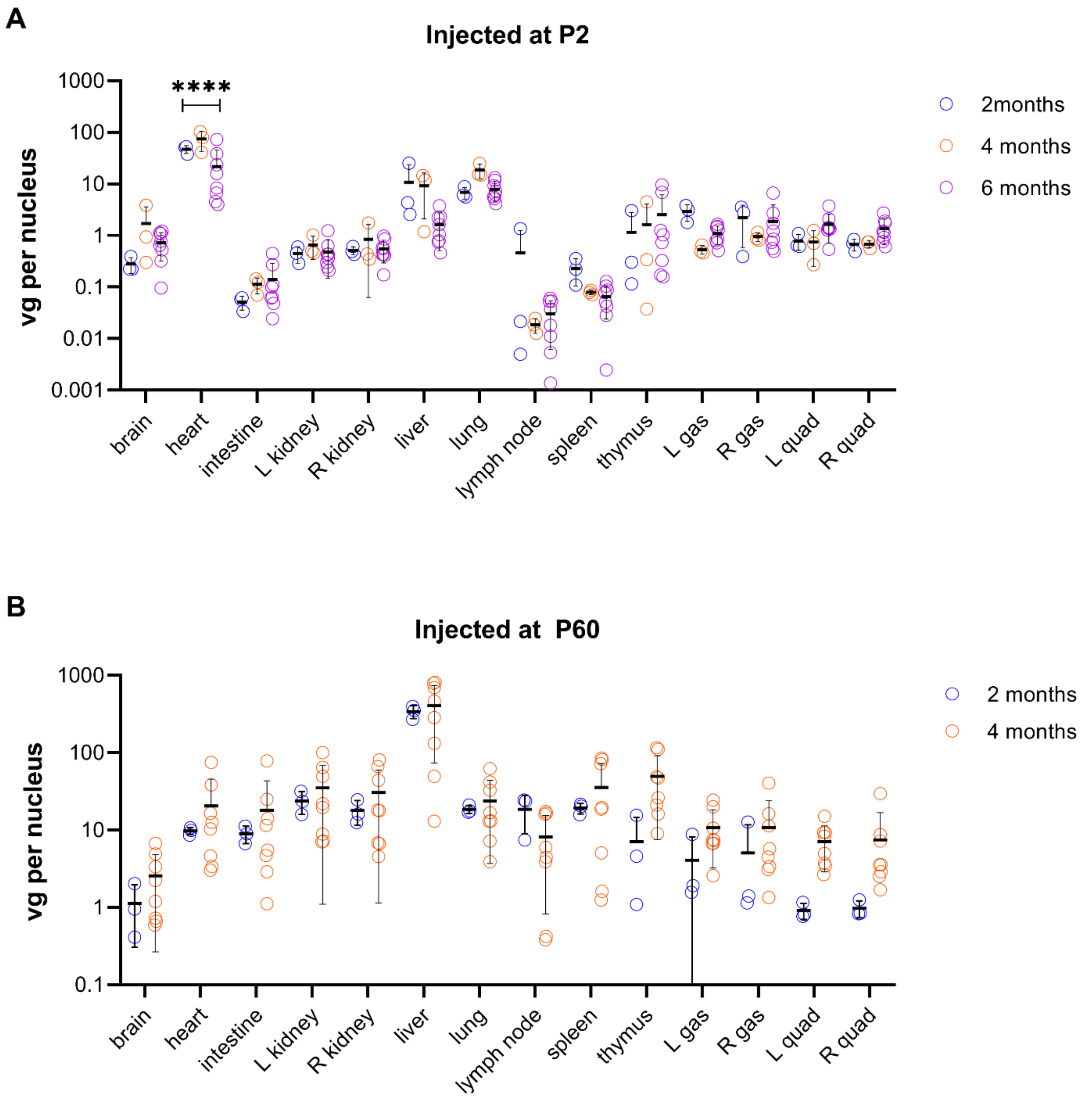

Figure S4. **AAV vector genomes are relatively stable over time.**

(A) Biodistribution of AAV in organs and tissues at 2-, 4-, and 6-months post-injection of *Lipa*$^{-/-}$ mice treated at P2. Change in vector genomes found in the heart is the only statistically significant change. **(B)** Biodistribution of AAV in organs and tissues at 2- and 4-months post-injection of *Lipa*$^{-/-}$ mice treated at P60. All data represented as mean ± SD (n=5-8). Statistical significance was defined as $p \leq 0.05$ (****$p \leq 0.0001$), using two-way ANOVA with Tukey's post-hoc test.

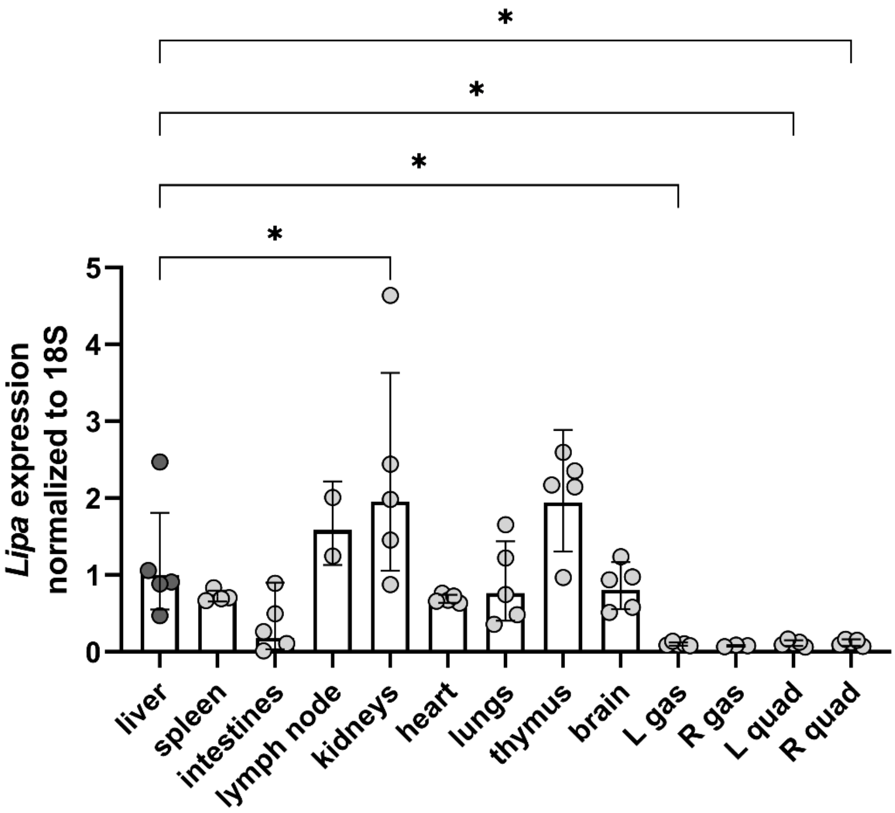

Figure S5. **Endogenous *Lipa* expression in various organs and tissues in WT mice.**

Lipa is expressed as relative to the liver. All data represented as mean ± SD (n=2-8). Statistical significance relative to liver expression was defined as p≤0.05 using one-way ANOVA with Tukey's post-hoc test.

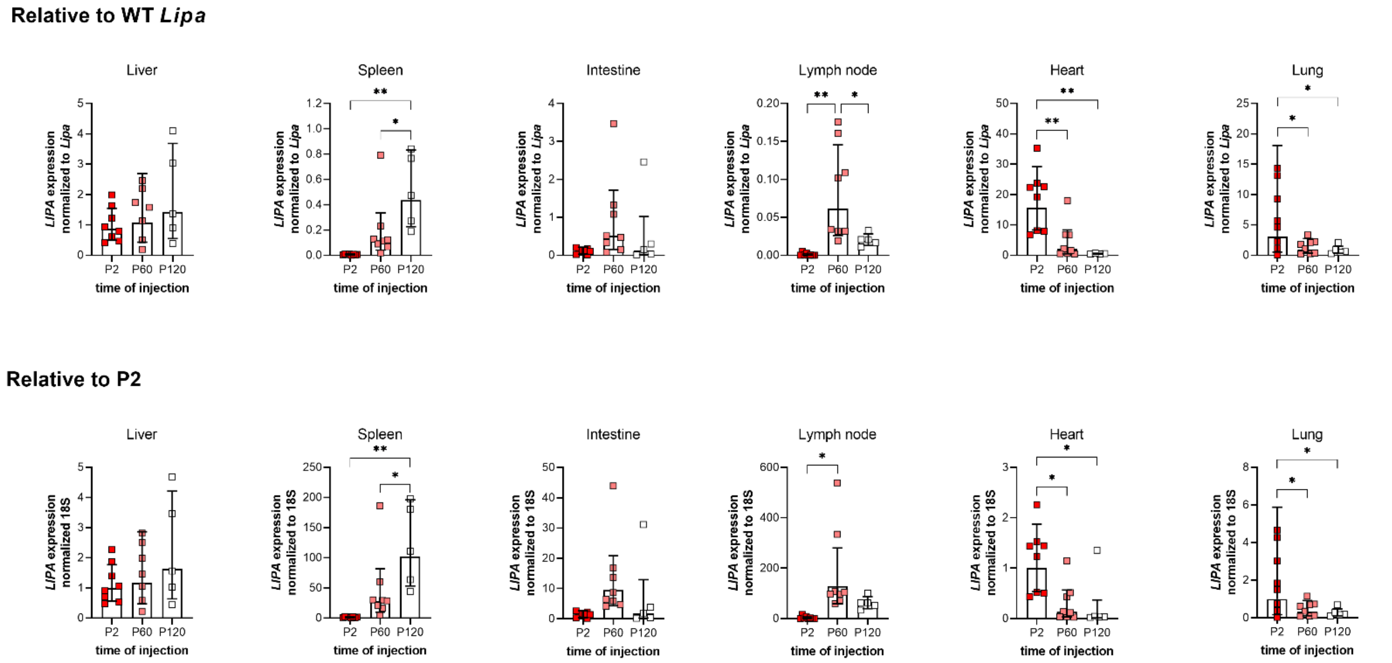

Figure S6. **RT-qPCR expression of the human *LIPA* transgene in treated mice at the 6 month endpoint.**

Expression data is presented as mRNA levels of human *LIPA* compared to the endogenous *Lipa* expression in WT, or compared to expression at when treated at P2. All data represented as mean ± SD (n=5-8). Statistical significance was defined as $p \leq 0.05$ (*$p \leq 0.05$, **$p \leq 0.01$), using one-way ANOVA with Tukey's post-hoc test.

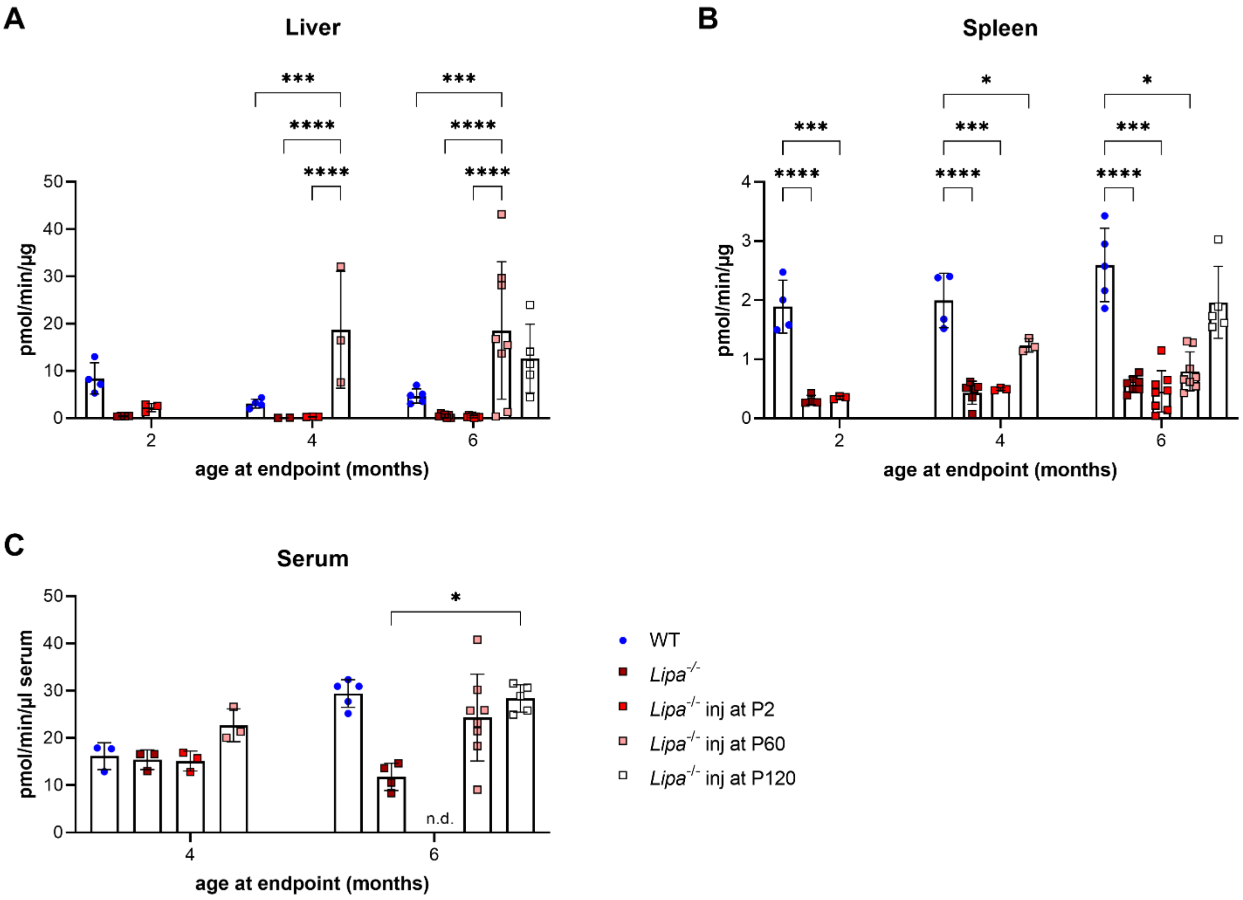

Figure S7. Lysosomal acid lipase activity is reduced in *Lipa*$^{-/-}$ mice at all timepoints.

Lysosomal acid lipase activity in **(A)** liver, **(B)** spleen, and **(C)** serum at 2, 4 and 6 months. Treatment at later time points (P60 and P120) result in restored enzyme activity in the liver. All data represented as mean ± SD (n=5-8). Statistical significance was defined as p≤0.05 (*p≤0.05, **p≤0.01, ***p≤0.001, ****p≤0.0001), using two-way ANOVA with Tukey’s post-hoc test. n.d. = not determined due to insufficient sample.

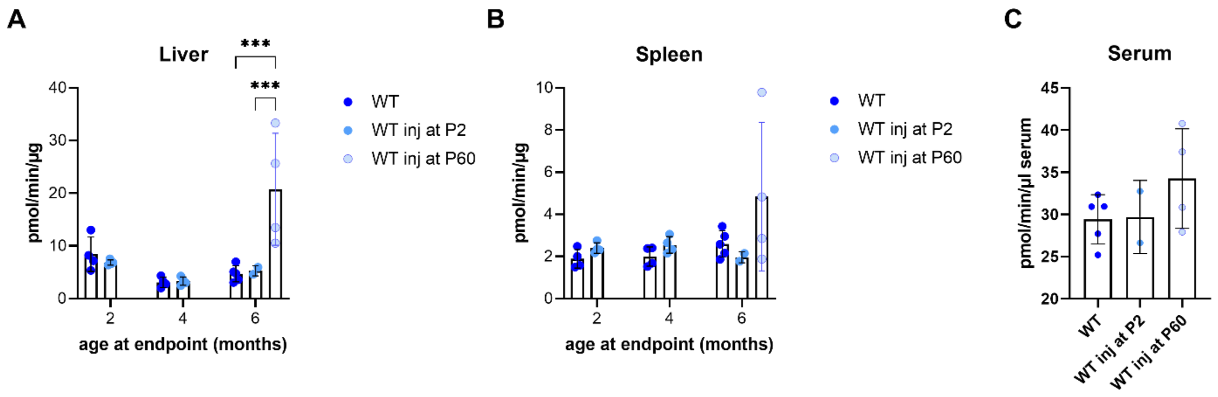

Figure S8. Lysosomal acid lipase activity in treated WT mice.

(A) Similar to treatment of *Lipa*$^{-/-}$ mice, only treatment at P60 resulted in statistically significant increase in LAL activity in liver. **(B)** Spleen LAL activity did not change with treatment in WT mice. **(C)** At the 6 month endpoint, serum LAL activity did not significantly change with treatment. Statistical significance was defined as $p \le 0.05$ (***$p \le 0.001$), using two-way ANOVA (A, B) or one-way ANOVA (C) with Tukey's post-hoc test.

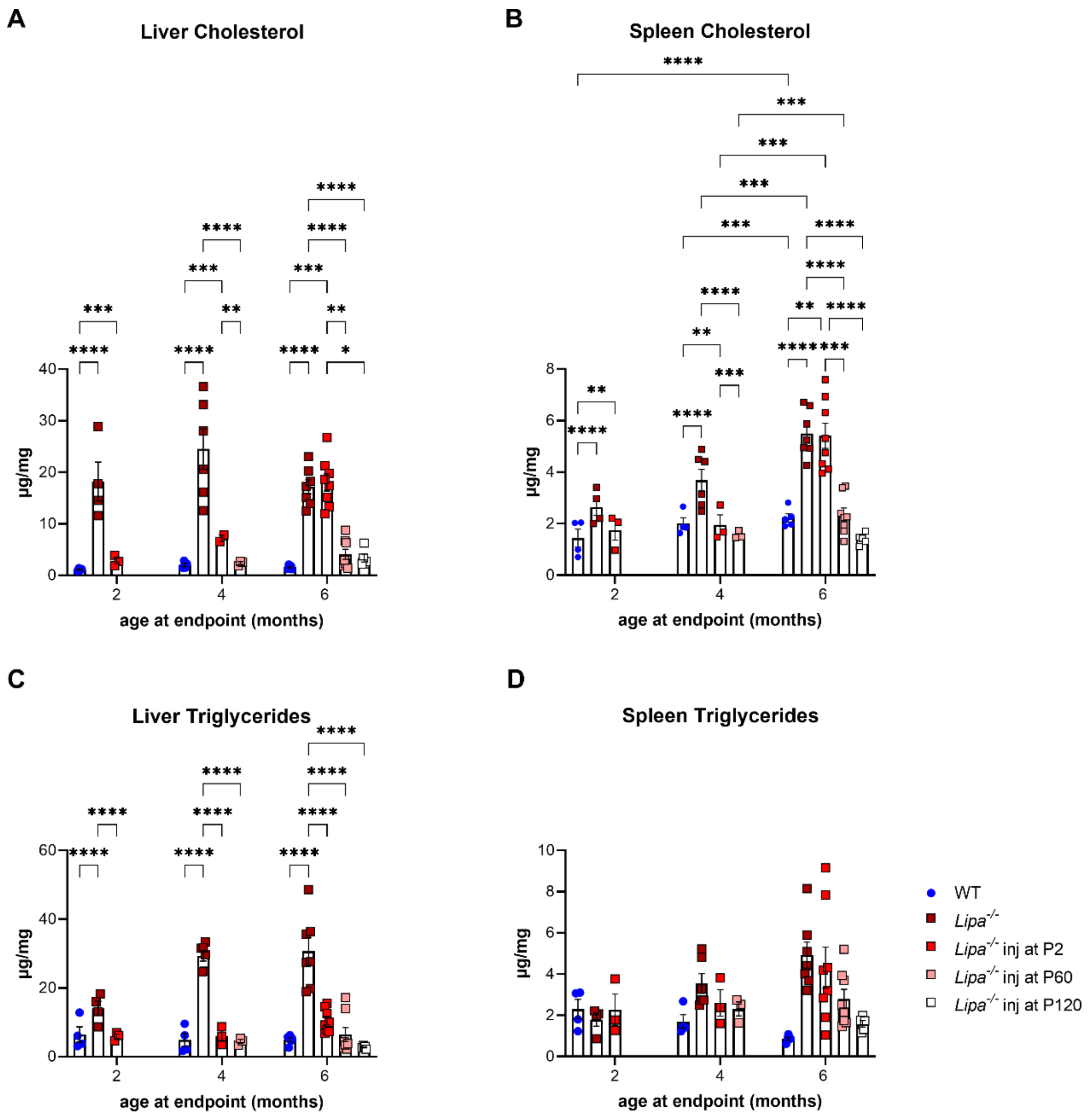

Figure S9. Cholesterol and triglyceride content at 2, 4, 6 months of age.

(A-B) Cholesterol content in (A) liver and (B) spleen at 2, 4, 6 months. **(C-D)** Triglyceride content in (C) liver and (D) spleen at 2, 4, 6 month endpoints. All data represented as mean ± SD (n=3-8). Statistical significance was defined as p≤0.05 (*p≤0.05, **p≤0.01, ***p≤0.001, ****p≤0.0001), using two-way ANOVA with Tukey's post-hoc test.

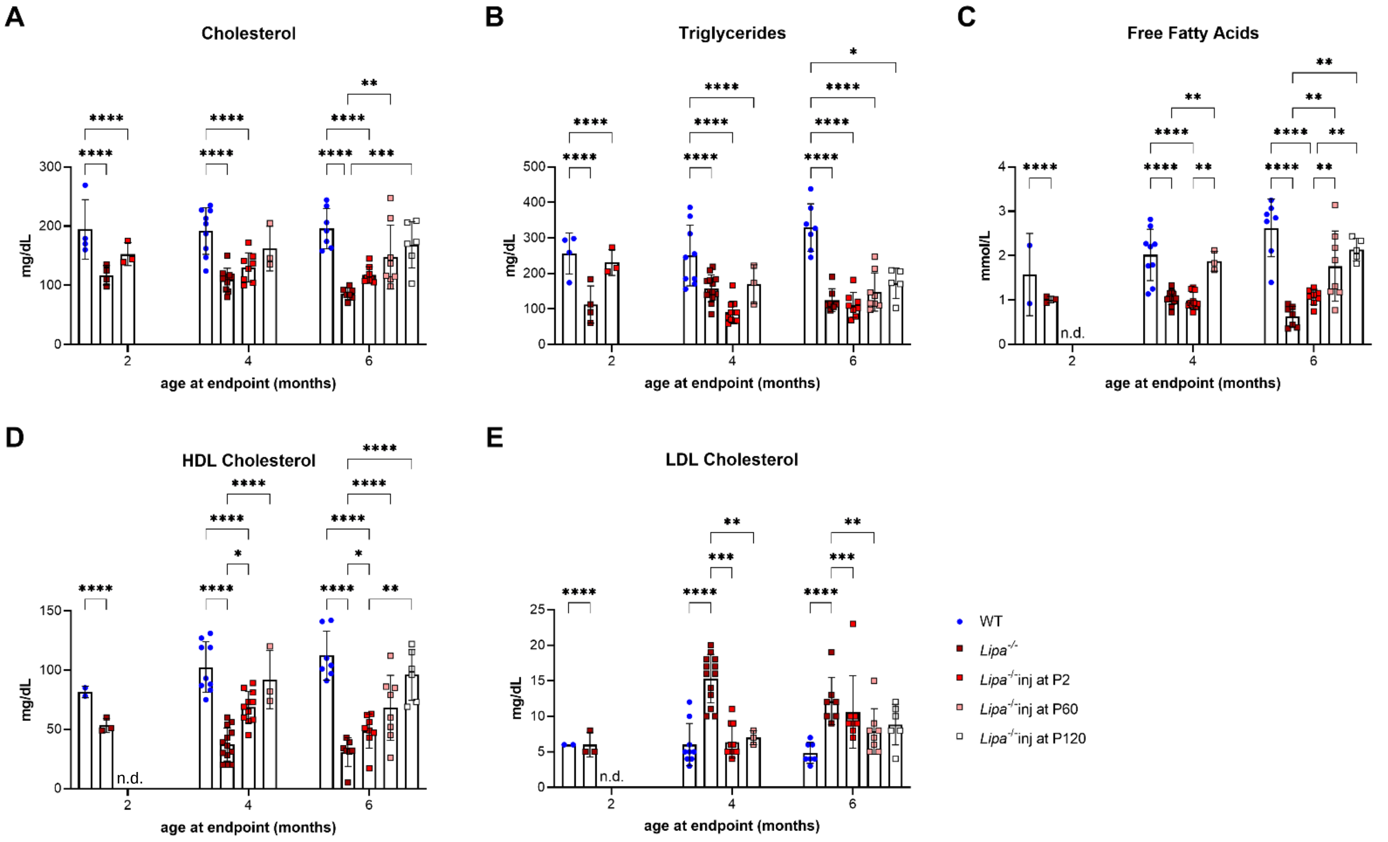

Figure S10. Serum lipid panel at 2, 4, and 6 months.

(A) Cholesterol, **(B)** Triglyceride, **(C)** free fatty acids **(D)** HDL cholesterol, **(E)** LDL cholesterol, and were measured in blood serum at 2, 4 and 6 months of age. All data represented as mean ± SD (n=5-8). Statistical significance was defined as p≤0.05 (*p≤0.05, **p≤0.01, ***p≤0.001, ****p≤0.0001), using two-way ANOVA with Tukey's post-hoc test. n.d. = not determined due to insufficient sample at the 2-month endpoint.

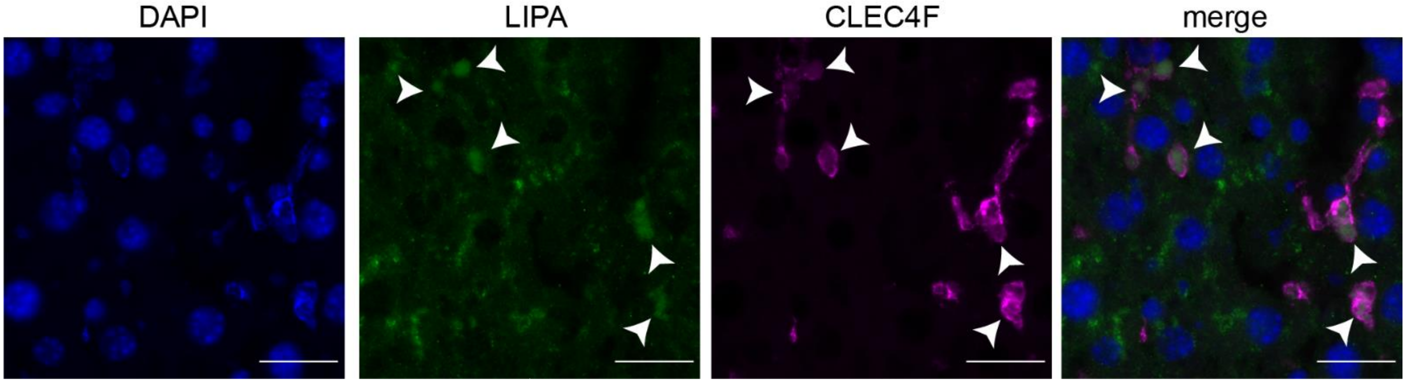

Figure S11. LIPA is expressed in hepatocytes and Kupffer cells.

Immunofluorescence staining of liver sections from WT mice treated at P60, 6 month endpoint. LIPA (green) is expressed throughout the liver. CLEC4F (magenta) is a Kupffer cell marker. The merged image shows co-localization of LIPA with CLEC45 (as indicated by arrows), as well as LIPA staining in hepatocytes (non-Kupffer cells). Scale bar = 25µm.

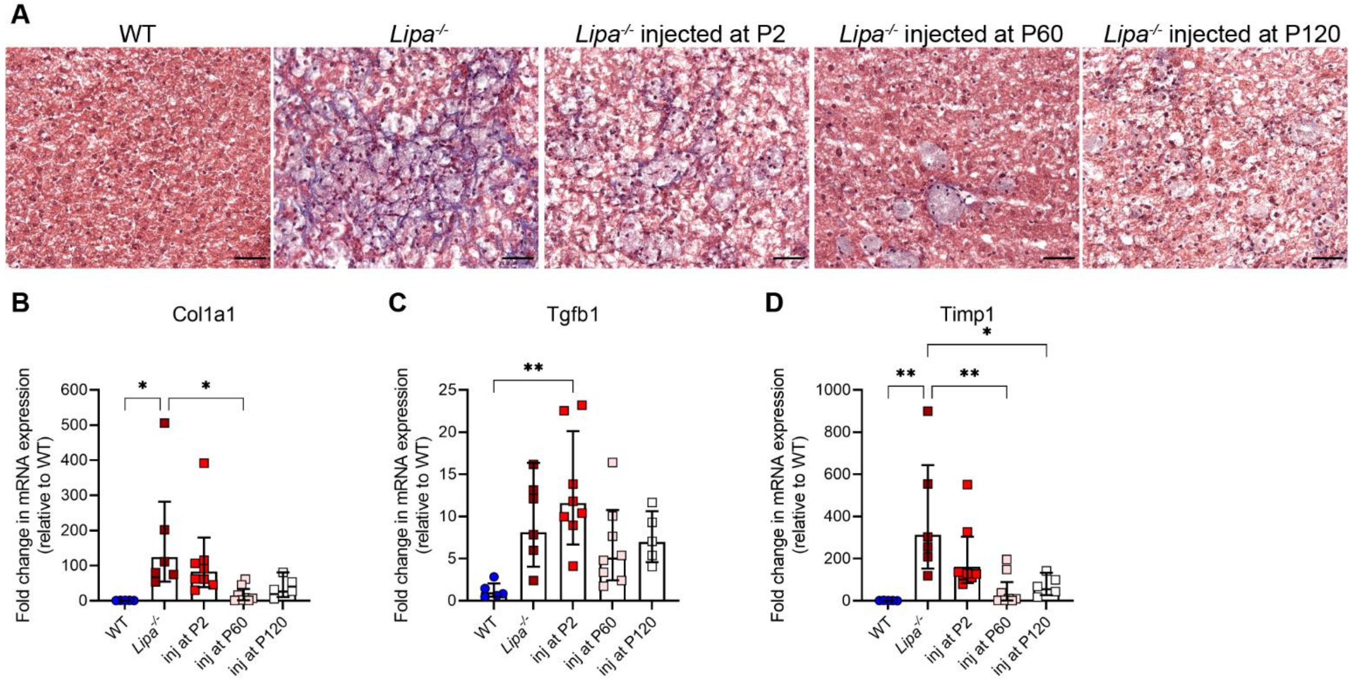

Figure S12. Fibrosis is decreased with treatment in *Lipa$^{-/-}$* mice.

(A) Masson's Trichrome staining of liver sections at the 6 month endpoint. *Lipa$^{-/-}$* mice show high amounts of fibrosis as evident from collagen staining (blue). Treatment at all timepoints decreases the amount of collagen staining. Scale bar = 25µm. **(B-D)** RT-qPCR was used to determine the expression of fibrosis genes (B) *Col1a1*, (C) *Tgfb1*, (D) *Timp1* in livers. All data represented as mean ± SD (n=2-8). Statistical significance was defined as p≤0.05 (*p≤0.05, **p≤0.01), using one-way ANOVA with Tukey's post-hoc test.

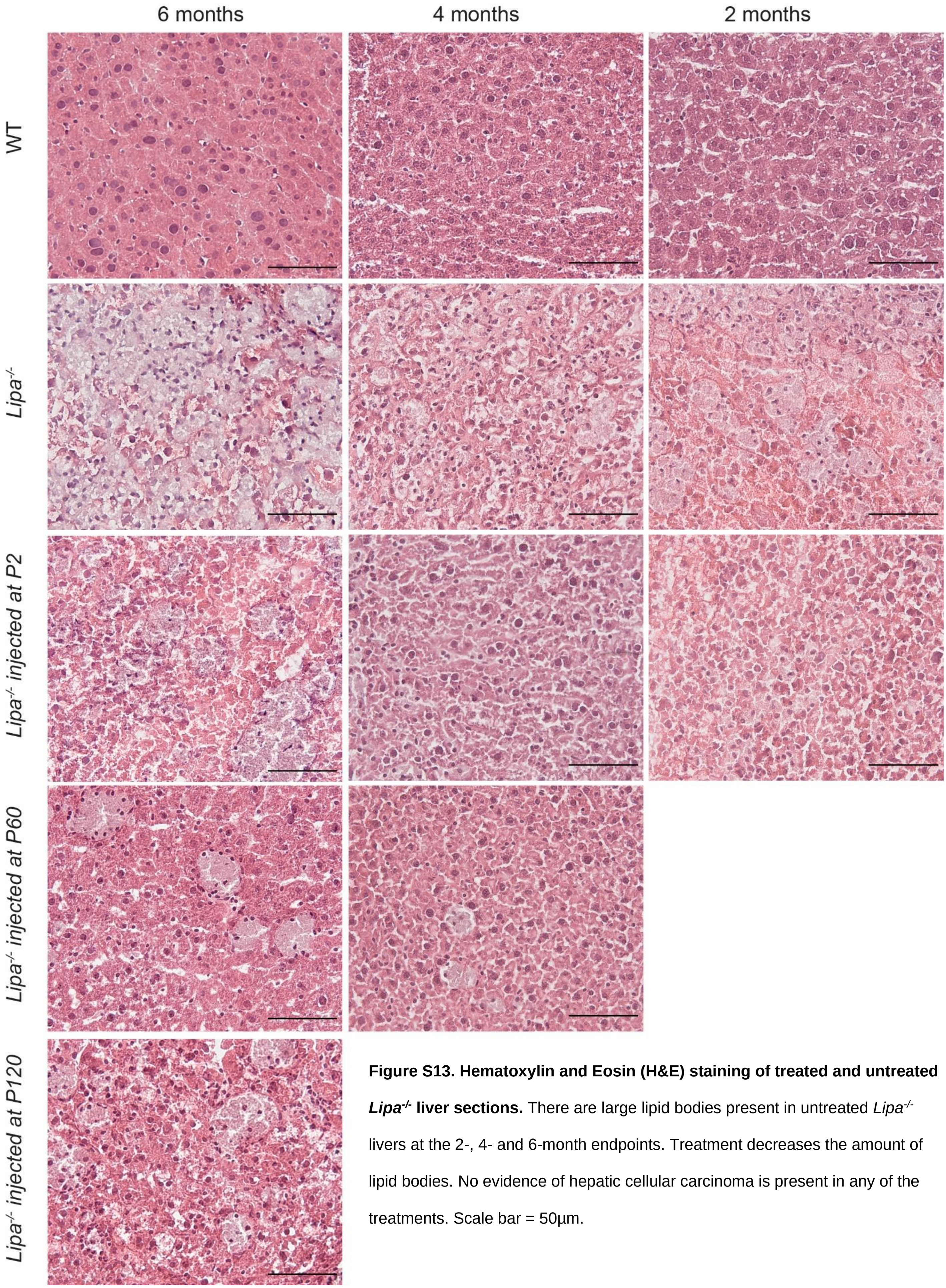

Figure S13. Hematoxylin and Eosin (H&E) staining of treated and untreated *Lipa*$^{-/-}$ liver sections. There are large lipid bodies present in untreated *Lipa*$^{-/-}$ livers at the 2-, 4- and 6-month endpoints. Treatment decreases the amount of lipid bodies. No evidence of hepatic cellular carcinoma is present in any of the treatments. Scale bar = 50µm.

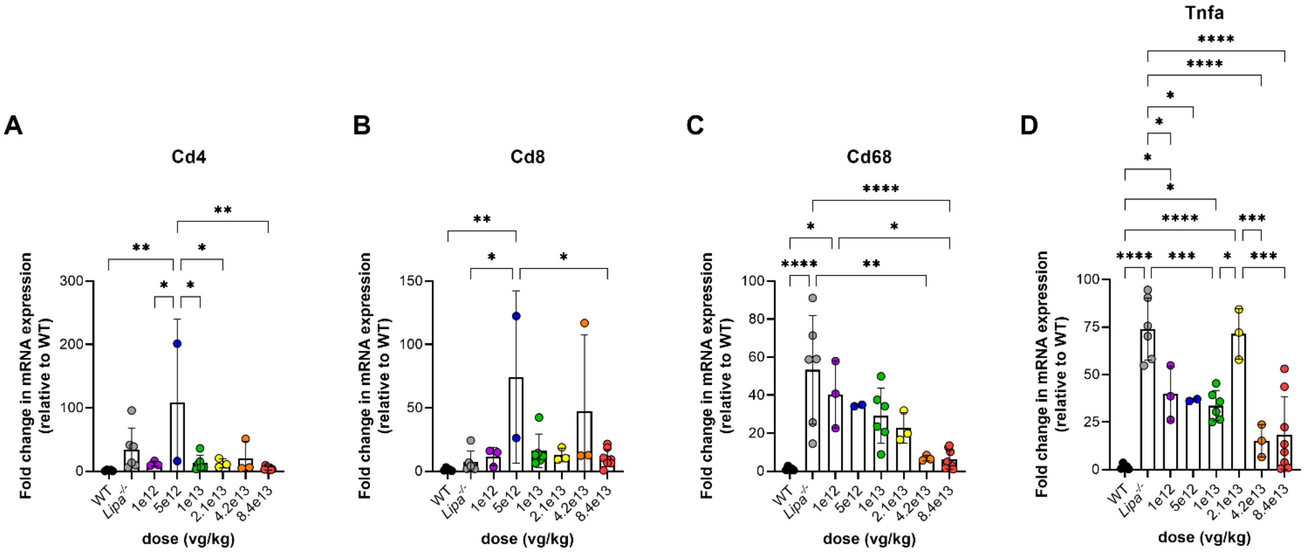

Figure S14. RT-qPCR analysis of various markers of inflammation in livers after different doses of gene therapy.

All data represented as mean ± SD (n=2-8). Statistical significance was defined as p≤0.05 (*p≤0.05, **p≤0.01, ***p≤0.001, ****p≤0.0001), using one-way ANOVA with Tukey's post-hoc test.

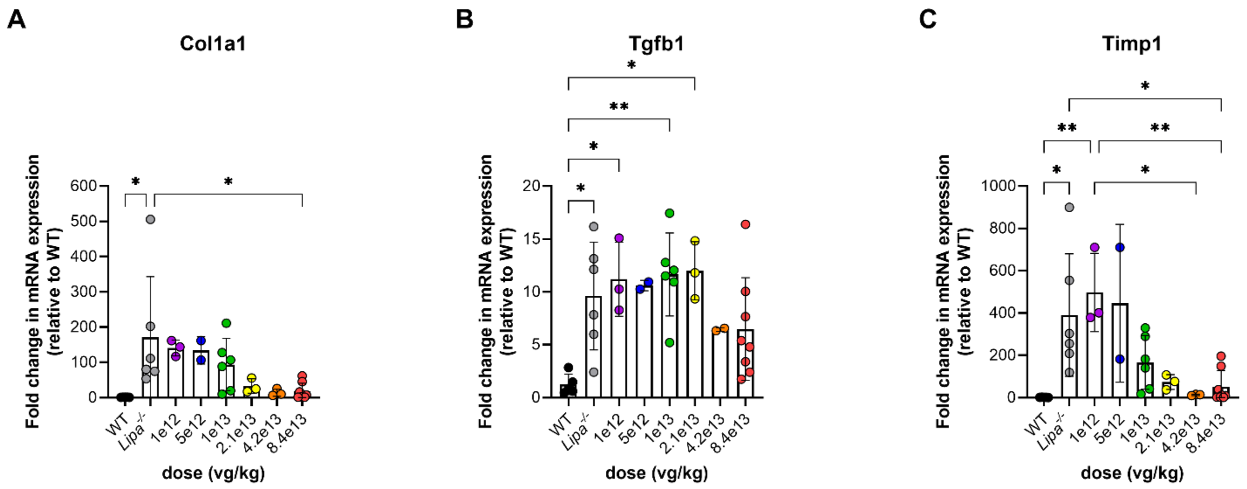

Figure S15. **RT-qPCR analysis of various markers of fibrosis after treatment with different doses.**

All data represented as mean ± SD (n=2-8). Statistical significance was defined as p≤0.05 (*p≤0.05, **p≤0.01), using one-way ANOVA with Tukey's post-hoc test.

Table S1. Biodistribution of AAV at 6-month endpoint. Values are represented as mean vg/nucleus ± SD.

	**Injected at P2 (n=8)**	**Injected at P60 (n=8)**	**Injected at P120 (n=5)**
**Brain**	1.20±0.14	2.55±0.81	1.64±0.21
**Heart**	21.82±8.41	20.52±8.79	12.9412.11
**Intestines**	0.14±0.05	17.93±9.08	12.58±3.73
**Liver**	1.62±0.39	406.13±117.59	90.69±21.48
**Lung**	7.87±1.16	23.91±7.15	45.47±14.73
**Lymph node**	0.03±0.01	8.14±2.59	4.99±1.94
**L kidney**	0.48±0.12	34.95±11.97	33.00±6.93
**R kidney**	0.55±0.09	30.66±10.44	38.83±8.58
**Spleen**	0.07±0.02	35.66±13.19	28.31±9.92
**Thymus**	2.52±1.28	49.44±14.81	19.25±8.36
**L gastrocnemius**	1.10±0.15	10.75±2.66	6.19±3.48
**R gastrocnemius**	1.86±0.73	10.79±4.61	11.89±5.07
**L quadricep**	1.65±0.33	7.11±1.48	4.55±0.65
**R quadricep**	1.38±0.25	7.50±3.29	6.81±1.53

Table S2. Biodistribution of AAV at the 4-month endpoint. Values are represented as mean vg/nucleus ± SD.

	**Injected at P2 (n=3)**	**Injected at P60 (n=3)**
**Brain**	1.70±1.09	1.13±0.48
**Heart**	74.34±18.01	0.97±0.61
**Intestines**	0.11±0.02	8.97±1.33
**Liver**	9.21±4.09	340.12±37.33
**Lung**	18.42±3.32	18.61±1.25
**Lymph node**	0.02±0.00	18.55±5.58
**L kidney**	0.66±0.18	23.74±4.53
**R kidney**	0.84±0.45	17.78±3.59
**Spleen**	0.08±0.01	19.26±1.79
**Thymus**	1.63±1.44	7.09±4.36
**L gastrocnemius**	0.54±0.06	4.09±2.35
**R gastrocnemius**	0.95±0.10	5.08±3.09
**L quadricep**	0.74±0.29	0.91±0.13
**R quadricep**	0.68±0.07	0.98±0.14

Table S3. Biodistribution of AAV at the 2-month endpoint. Values are represented as mean vg/nucleus ± SD.

	**Injected at P2 (n=3)**
**Brain**	0.28±0.06
**Heart**	47.27±4.76
**Intestines**	0.05±0.01
**Liver**	10.73±7.29
**Lung**	6.85±1.01
**Lymph node**	0.46±0.45
**L kidney**	0.45±0.09
**R kidney**	0.51±0.05
**Spleen**	0.23±0.07
**Thymus**	1.15±0.95
**L gastrocnemius**	2.92±0.60
**R gastrocnemius**	2.23±0.95
**L quadricep**	0.78±0.15
**R quadricep**	0.67±0.10

Table S4. Biodistribution of AAV at the various doses, treated at P60, 6 month endpoint. Values represent mean vg/nucleus ± SD.

	**$1.0x10^{12}$ vg/kg (n=3)**	**$5.0x10^{12}$ vg/kg (n=2)**	**$1.0x10^{13}$ vg/kg (n=3)**	**$2.1x10^{13}$ vg/kg (n=3)**	**$4.2x10^{13}$ vg/kg (n=3)**	**$8.4x10^{13}$ vg/kg (n=8)**
**Brain**	0.03±0.01	0.32±0.12	0.23±0.06	0.77±0.17	1.46±0.62	2.55±0.81
**Heart**	0.18±0.03	0.87±0.33	1.80±0.65	3.70±2.38	12.51±1.55	20.52±8.79
**Intestines**	0.21±0.05	0.88±0.08	1.94±0.61	4.29±2.21	10.72±3.26	17.93±9.08
**Liver**	0.03±0.00	1.58±0.06	10.84±5.88	39.99±12.80	316.38±95.55	406.13±117.59
**Lung**	0.15±0.02	0.89±0.53	2.28±0.84	3.30±0.15	23.89±8.48	23.91±7.15
**Lymph node**	0.03±0.00	0.93±0.68	0.38±0.11	0.75±0.37	5.32±4.45	8.14±2.59
**L kidney**	0.11±0.02	1.49±0.67	3.98±2.15	10.94±4.56	24.38±3.99	34.95±11.97
**R kidney**	0.14±0.04	1.92±0.16	4.39±1.99	9.23±2.65	44.94±10.31	30.66±10.44
**Spleen**	0.02±0.00	0.31±0.21	1.13±0.48	3.24±1.32	33.41±12.28	35.66±13.19
**Thymus**	0.22±0.11	0.82±0.15	3.08±1.13	6.73±1.84	74.99±2.58	49.44±14.81
**L gastrocnemius**	0.12±0.02	1.47±0.96	0.92±0.20	2.66±1.21	6.97±2.76	10.75±2.66
**R gastrocnemius**	0.36±0.19	1.65±1.21	1.19±0.40	2.54±0.73	12.25±4.62	10.79±4.61
**L quadricep**	0.15±0.03	1.09±0.73	0.64±0.12	1.54±0.61	9.26±3.41	7.11±1.48
**R quadricep**	0.10±0.02	0.27±0.11	0.66±0.14	1.37±0.67	6.18±4.14	7.50±3.29

Table S5. Sequences of primers used in this study

		Sequence
WT – 323 bp	exon5-F	5'- CTGCATGGAGACTCACAAAGGA- 3'
	exon5-R	5'- AAGTCTTCCCTGTTCCCATGG- 3'
LAL KO – 203 bp	HPRT-F	5'- CGTCGTGATTAGCGATGATGA- 3'
	HPRT-R	5'- TCCAGCAGGTCAGCAAAGAA-3'
AAV biodistribution	Primer1	5'-TGACGTCAATGGGAGTTTGTT-3'
	Primer2	5'-ATATAGACCTCCCACCGTACAC-3'
	Probe	5'-/56-FAM/CATTGACGC/ZEN/AAATGGGCGGTAGG/3IABkFQ/-3'
Human *LIPA* expression	Primer1	5'-ACTAGAATCTGCCAGCAAGC-3'
	Primer2	5'-TCTGTGCCTTAACCGAATTCC-3'
	Probe	5'-/56-FAM/TCCCAAACC/ZEN/AGTTGTCTTCCTGCA/3IABkFQ/-3'
Mouse *Lipa* expression	Primer1	5'-ATTCTCAAGGCTGCACCATAG-3'
	Primer2	5'-CAAGCGTCCCAATTGAAGTAG-3'
	Probe	5'-/56-FAM/TTAGTCTTG/ZEN/GCTCCCGTGTTGTCTC/3IABkFQ/-3'
Eukaryotic 18S rRNA Endogenous Control		ThermoFisher Cat# 4319413E
Pre-designed Taqman Gene Expression Assays (ThermoFisher)		
**Gene**		**Assay ID**
Cd4		Mm00442754_m1
Cd8a		Mm01182108_m1
Cd68		Mm00839636_m1
Tnfa		Mm99999068_m1
Col1a1		Mm00801666_g1
Tgfb1		Mm01178820_m1
Timp1		Mm00441818_m1

Table S6. Complete sequence of the therapeutic cassette (miniCMV promoter-*hLIPA*-SV40 polyA) including the AAV2 ITR and truncated ITR region.

```
CTGCGCGCTCGCTCGCTCACTGAGGCCGCCCGGGCAAAGCCCGGGCGTCGGGCGACCTTTGGTCGCCCGGCCTCAGTGAGCGAGCGAGCGCGCAGAG
AGGGAGTGGCCAACTCCATCACTAGGGGTTCCTAGGAAGCTTTCGTTACATAACTTACGGTAAATGGCCCGCCTGGCTGACCGCCCAACGACCCCCG
GACTCACGGGGATTTCCAAGTCTCCACCCCATTGACGTCAATGGGAGTTTGTTTTGGCACCAAAATCAACGGGACTTTCCAAAATGTCGTAACAACT
CCGCCCCATTGACGCAAATGGGCGGTAGGCGTGTACGGTGGGAGGTCTATATAAGCAGAGCTCGTTTAGTGAACCGCTCGACCGGGGTACCCGGCCG
GCTAGCCGCCACCATGAAAATGCGGTTCTTGGGGTTGGTGGTCTGTTTGGTTCTCTGGACCCTGCATTCTGAGGGGTCTGGAGGGAAACTGACAGCT
GTGGATCCTGAAACAAACATGAATGTGAGTGAAATTATCTCTTACTGGGGATTCCCTAGTGAGGAATACCTAGTTGAGACAGAAGATGGATATATTC
TGTGCCTTAACCGAATTCCTCATGGGAGGAAGAACCATTCTGACAAAGGTCCCAAACCAGTTGTCTTCCTGCAACATGGCTTGCTGGCAGATTCTAG
TAACTGGGTCACAAACCTTGCCAACAGCAGCCTGGGCTTCATTCTTGCTGATGCTGGTTTTGACGTGTGGATGGGCAACAGCAGAGGAAATACCTGG
TCTCGGAAACATAAGACACTCTCAGTTTCTCAGGATGAATTCTGGGCTTTCAGTTATGATGAGATGGCAAAATATGACCTACCAGCTTCCATTAACT
TCATTCTGAATAAAACTGGCCAAGAACAAGTGTATTATGTGGGTCATTCTCAAGGCACCACTATAGGTTTTATAGCATTTTCACAGATCCCTGAGCT
GGCTAAAAGGATTAAAATGTTTTTTGCCCTGGGTCCTGTGGCTTCCGTCGCCTTCTGTACTAGCCCTATGGCCAAATTAGGACGATTACCAGATCAT
CTCATTAAGGACTTATTTGGAGACAAAGAATTTCTTCCCCAGAGTGCGTTTTTGAAGTGGCTGGGTACCCACGTTTGCACTCATGTCATACTGAAGG
AGCTCTGTGGAAATCTCTGTTTTCTTCTGTGTGGATTTAATGAGAGAAATTTAAATATGTCTAGAGTGGATGTATATACAACACATTCTCCTGCTGG
AACTTCTGTGCAAAACATGTTACACTGGAGCCAGGCTGTTAAATTCCAAAAGTTTCAAGCCTTTGACTGGGGAAGCAGTGCCAAGAATTATTTTCAT
TACAACCAGAGTTATCCTCCCACATACAATGTGAAGGACATGCTTGTGCCGACTGCAGTCTGGAGCGGGGGTCACGACTGGCTTGCAGATGTCTACG
ACGTCAATATCTTACTGACTCAGATCACCAACTTGGTGTTCCATGAGAGCATTCCGGAATGGGAGCATCTTGACTTCATTTGGGGCCTGGATGCCCC
TTGGAGGCTTTATAATAAAATTATTAATCTAATGAGGAAATATCAGTGAGCATGCACTAGTGCGGCCGCGGATCTCAGACATGATAAGATACATTGA
TGAGTTTGGACAAACCACAACTAGAATGCAGTGAAAAAAATGCTTTATTTGTGAAATTTGTGATGCTATTGCTTTATTTGTAACCATTATAAGCTGC
AATAAACAAGTTAACAACAACAATTGCATTCATTTTATGTTTCAGGTTCAGGGGGAGGTGTGGGAGGTTTTTTAGGTTTAAACCCCACTCCCTCTCT
GCGCGCTCGCTCGCTCACTGAGGCCGGGCGACCAAAGGTCGCCCGACGCCCGGGCTTTGCCCGGGCGGCCTCAGTGAGCGAGCGAGCGCGCAG
```
